# Crosstalk between purinergic receptor P2Y_11_ and chemokine receptor CXCR7 is regulated by CXCR4 in human macrophages

**DOI:** 10.1007/s00018-024-05158-7

**Published:** 2024-03-13

**Authors:** Dominik Klaver, Hubert Gander, Beatrice Frena, Marco Amato, Martin Thurnher

**Affiliations:** 1 Immunotherapy Unit, Department of Urology, Medical University of Innsbruck, Innrain 66a, Innsbruck, 6020 Austria; 2 Central Institute for Blood Transfusion & Department of Immunology (ZIB), Tirol Kliniken GmbH, Innsbruck, Austria

**Keywords:** P2Y_11_, CXCR4, PDE4, Cyclic AMP, CXCR7, EGFR, CCL20

## Abstract

P2Y_11_ is a G protein-coupled ATP receptor that activates IL-1 receptor (IL-1R) in a cyclic AMP dependent manner. In human macrophages, P2Y_11_/IL-1R crosstalk with CCL20 as a prime target is controlled by phosphodiesterase 4 (PDE4), which mediates breakdown of cyclic AMP. Here, we used gene expression analysis to identify activation of CXCR4 and CXCR7 as a hallmark of P2Y_11_ signaling. We found that PDE4 inhibition with rolipram boosts P2Y_11_/IL-1R-induced upregulation of CXCR7 expression and CCL20 production in an epidermal growth factor receptor dependent manner. Using an astrocytoma cell line, naturally expressing CXCR7 but lacking CXCR4, P2Y_11_/IL-1R activation effectively induced and CXCR7 agonist TC14012 enhanced CCL20 production even in the absence of PDE4 inhibition. Moreover, CXCR7 depletion by RNA interference suppressed CCL20 production. In macrophages, the simultaneous activation of P2Y_11_ and CXCR7 by their respective agonists was sufficient to induce CCL20 production with no need of PDE4 inhibition, as CXCR7 activation increased its own and eliminated CXCR4 expression. Finally, analysis of multiple CCL chemokines in the macrophage secretome revealed that CXCR4 inactivation and CXCR7 activation selectively enhanced P2Y_11_/IL-1R-mediated secretion of CCL20. Altogether, our data establish CXCR7 as an integral component of the P2Y_11_/IL-1R-initiated signaling cascade and CXCR4-associated PDE4 as a regulatory checkpoint.

**Supplementary Information:**

The online version contains supplementary material available at 10.1007/s00018-024-05158-7.

## Introduction

Chemokine receptors are seven-transmembrane proteins that mediate cell trafficking but may also regulate cell activation, proliferation, cell death and survival [1]. Classical chemokine receptors are G protein-coupled receptors (GPCR), which govern chemokine-directed cell migration, while atypical chemokine receptors (ACKR) can act as chemokine scavengers or exhibit effects through other signaling pathways [1].

Within the large family of chemokine receptors, CXCR4 and CXCR7 maintain a close relationship [2]. CXCR4, originally identified as co-receptor during HIV infection, plays a major role in steady state homeostatic processes. CXCR4 for instance mediates B cell positioning in the dark zone of germinal centers [3] as well as the retention of hematopoietic stem cells in the bone marrow [4]. CXCR4 antagonist plerixafor, a bicyclam also known as AMD3100, thus acts as a potent stem cell mobilizer in the treatment of non-Hodgkin’s lymphoma or multiple myeloma [5]. CXCR4 is also required for lymphangiogenesis [6] and has been reported to trans-repress genes involved in immune activation [7].

While chemotactic functions are obviously ligand-dependent, CXCR4 may also exhibit some degree of ligand-independent, i.e. constitutive activity [8–10]. CXCR4 mainly engages the Gα_i_ subunit [11]. Upon dissociation from the Gαßγ heterotrimeric complex, Gα_i_ inhibits adenylyl cyclase (AC) activity and thus prevents cyclic AMP formation. Apart from classical G protein signaling, CXCR4 has also been shown to induce β-arrestin-mediated signaling. β-arrestins are not only involved in the termination of GPCR signaling and GPCR internalization, they may also contribute to the activation of other signaling pathways. β-arrestin-mediated signaling effects include the scaffolding for phosphodiesterase 4 [12], which hydrolyzes cyclic AMP. Thus, CXCR4 may act as a perfect sentinel controlling the levels of cyclic AMP by suppressing its formation as well as by facilitating its degradation.

CXCR7, also known as RDC-1, is an atypical chemokine receptor (ACKR3), which is non-chemotactic [13]. CXCR7 is upregulated by inflammatory cytokines such as IL-1β [14]. CXCR7 cannot couple to G-proteins, but instead interacts with β-arrestin to promote MEK/ERK signaling [15]. CXCR7 forms heterodimers with CXCR4 in a constitutive manner [16]. Accordingly, preformed CXCR4/CXCR7 heterodimers have been detected at the cell surface even in the absence of ligand.

CXCR7 may act as a negative regulator of CXCR4. Heterodimerization with CXCR7 selectively prevents CXCR4 from activating Gα_i_ proteins [16]. However, the CXCR4-CXCR7 complex constitutively recruits β-arrestin [17]. CXCR7 overexpression has been shown to be sufficient to reduce CXCL12-induced β-arrestin recruitment to CXCR4 and a CXCR7 agonist promoted the internalization of CXCR4 [18]. CXCR7 exerts various tissue protective and anti-thrombotic effects through a wide range of cells [19]. Administration of the CXCR7 agonist TC14012 after lung injury for instance promotes alveolar repair and reduces fibrosis [20]. However, malignant cells may exploit the protective effects of CXCR7 to promote tumor progression. CXCR7 is highly expressed in human glioma cells and mediates resistance to drug-induced apoptosis [21]. Likewise, CXCR7 is frequently upregulated in aggressive cancers, including therapy-resistant neuroendocrine prostate cancer [22].

P2Y_11_ is a class A GPCR that translates the danger signal ATP into adaptive responses [23, 24]. Among its striking features are the dual coupling to G_q_ and G_s_ proteins as well as its absence in rodents [25], what made corresponding animal models impossible. Moreover, the systematic examination of P2Y_11_ expression, activation and downstream signaling pathways has long been slowed down by the limited availability of pharmacology and immunology research tools.

In earlier studies, we and others have shown that P2Y_11_ is upregulated during differentiation of human M2 macrophages, both at the mRNA [26] and at the protein level [27]. Our finding in 2019 that P2Y_11_ protein is upregulated during M2 macrophage polarization [27] was the first demonstration of P2Y_11_ regulation at the protein level. In accordance with P2Y_11_‘s ability to increase the level of intracellular cyclic AMP via G_s_-mediated AC activation [28] and with the known cyclic AMP dependence of IL-1 signaling [29], we demonstrated both, in primary macrophages and in recombinant astrocytoma cells, that P2Y_11_ upregulates IL-1R in a cyclic AMP-dependent manner thus establishing P2Y_11_/IL-1R crosstalk [30, 31].

P2Y_11_ can act as a perfect sentinel of TNFα-induced inflammation: P2Y_11_ engages cyclic AMP signaling to suppress TNFα production and, at the same time, promotes the release (shedding) of soluble TNF receptors via ADAM17 to neutralize TNFα in the microenvironment [30]. Another hallmark of P2Y_11_ anti-inflammatory signaling in our studies was the transcriptional inactivation of all NLRP3 inflammasome components [31]. The P2Y_11_-induced production of vascular endothelial growth factor (VEGF) indicated that its cytoprotective function also included pro-angiogenic features. Moreover, in these studies transcriptomic and secretomic analyses of both, recombinant and native P2Y_11_ receptors, identified the multifunctional chemokine CCL20 as a prime target of P2Y_11_/IL-1R crosstalk.

In the present study, gene expression analysis was used to identify upregulation of CXCR4 and CXCR7 as hallmarks of a highly selective change in the chemokine receptor profile of macrophages in response to P2Y_11_ activation. We subsequently demonstrate that a highly selective chemokine response induced by P2Y_11_/IL-1R crosstalk is mediated by CXCR7 and regulated by CXCR4.

## Results

### P2Y_11_/IL-1R crosstalk upregulates CXCR7 in macrophages

The activity of 20 chemokine receptor genes including CCRs, CXCRs and atypical chemokine receptors was examined using NanoString technology in human monocyte-derived M2 macrophages (Fig. S1). Intriguingly, atypical chemokine receptor 3 (ACKR3), also known as CXCR7 or RDC-1, as well as CXCR4, also known as fusin and co-receptor for HIV entry [32], emerged as hallmarks of P2Y_11_-induced remodeling of the chemokine receptor profile in M2 macrophages (Table S1). CXCR7 was outstanding, because of its strong activation in response to cyclic AMP accumulation mediated by PDE4 inhibition with rolipram [33]. The boosting effect of rolipram in CXCR7 upregulation was reminiscent of the effect rolipram had in potentiating CCL20 production [31]. Of note, the atypical scavenging receptor ACKR4, which preferentially internalizes CCL20 [34], was downregulated, although not significantly (Table S1), altogether raising the possibility that CXCR7 participates in the regulation of CCL20 expression.

**Fig. 1:**
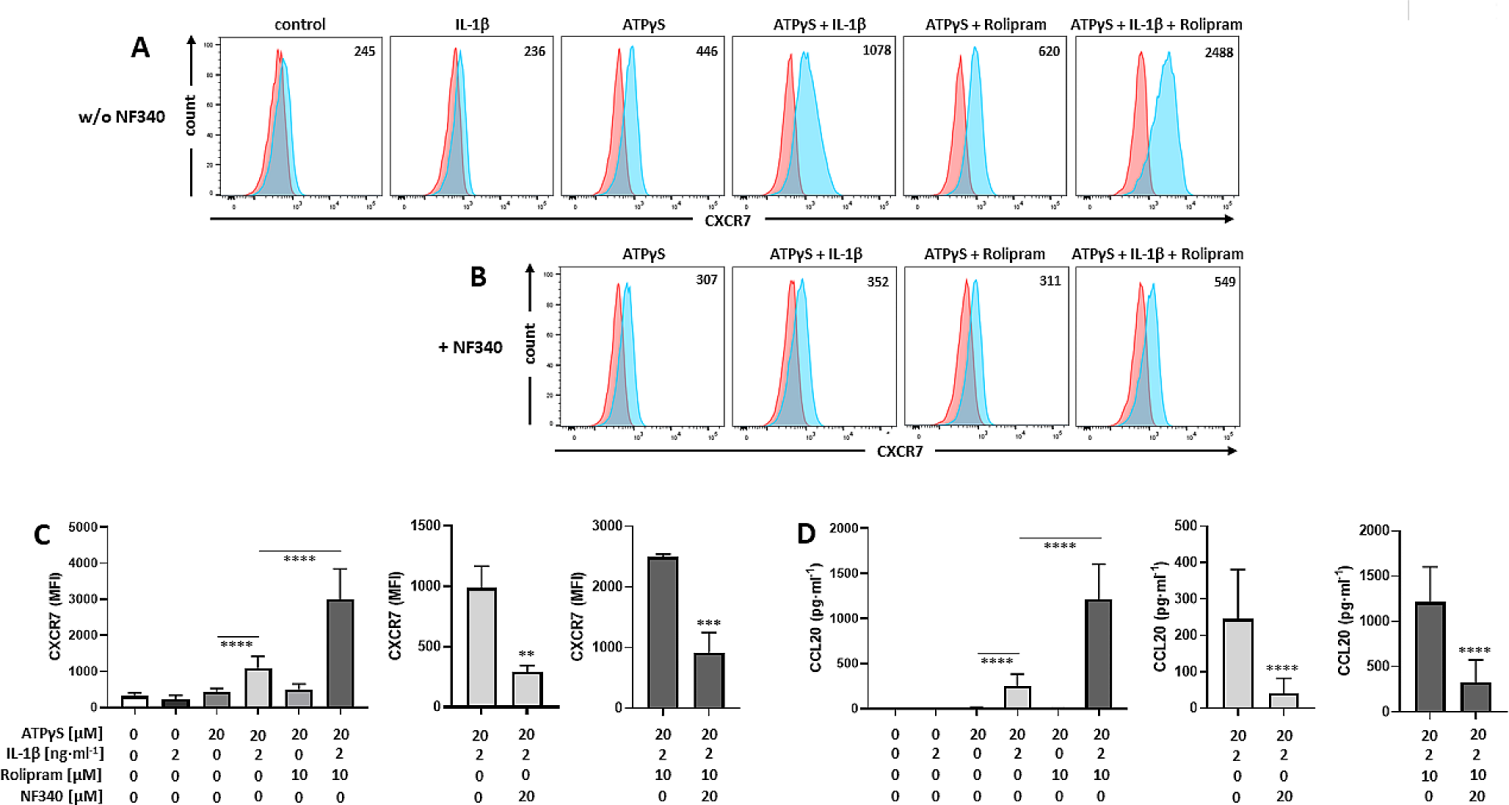
P2Y_11_ and IL-1R cooperate to upregulate CXCR7 surface expression and CCL20 production in primary human M2 macrophages: potentiation by PDE4 inhibition. **a** M2 macrophages were treated for 24 h with ATPγS (20 µM) alone or in combination with IL-1ß (2 ng/ml) in the presence or absence of rolipram (10 µM). Flow cytometry was used to determine CXCR7 expression (light blue; isotype controls in red). Numbers are mean fluorescence intensities (MFIs) after subtraction of isotype control MFIs. **b** P2Y_11_ receptor antagonist NF340 (20 µM) served to confirm that ATPγS-mediated changes were specific to P2Y_11_ receptor activation. **c** Quantification of CXCR7 expression (*n* = 3): mean values ± SD are shown. **p < 0.01, ***p < 0.001, ****p < 0.0001. D) Quantification of CCL20 production after the same treatments that were used to upregulate CXCR7 expression (*n* = 5). Mean values ± SD are shown. ****p < 0.0001

While P2Y_11_ agonist ATPγS and PDE4 inhibitor rolipram activated CXCR7 gene transcription in a synergistic manner, strong CXCR7 protein expression additionally required co-stimulation with IL-1ß (Fig. 1A/C). IL-1ß, which is known to co-stimulate its own mRNA and protein expression [35], has been shown to similarly serve as a second signal in P2Y_11_-driven CCL20 production [31]. Accordingly, P2Y_11_/IL-1R-induced and PDE4 inhibition enhanced both, CXCR7 protein expression and CCL20 secretion (Fig. 1C/D). In the signaling cascade leading from P2Y_11_ activation down to CCL20 production, P2Y_11_ activity is obviously sufficient to induce mRNA expression, but additionally requires IL-1R stimulation to induce protein expression. This applies to the IL-1R itself as well as to CXCR7 and CCL20. IL-1ß thus serves as a second, co-stimulatory signal that activates mRNA translation of these genes [35]. Importantly, the concomitant upregulation of CXCR7 and CCL20 was prevented by P2Y_11_ antagonist NF340 (Fig. 1B-D), which is currently the most useful antagonist at the P2Y_11_ receptor [30, 31, 36].

### P2Y_11_/IL-1R crosstalk transactivates the epidermal growth factor receptor to upregulate CXCR7 expression and CCL20 production

GPCR-mediated transactivation of the epidermal growth factor receptor (EGFR) is well established [37] although it has so far not been demonstrated for P2Y_11_. In addition, a ligand-independent role of CXCR7 in EGFR activation has been described [38, 39]. Moreover, EGFR signaling has been shown to promote CCL20 production in a variety of cancers [40, 41] as well as in keratinocytes from psoriasis patients [42].

**Fig. 2:**
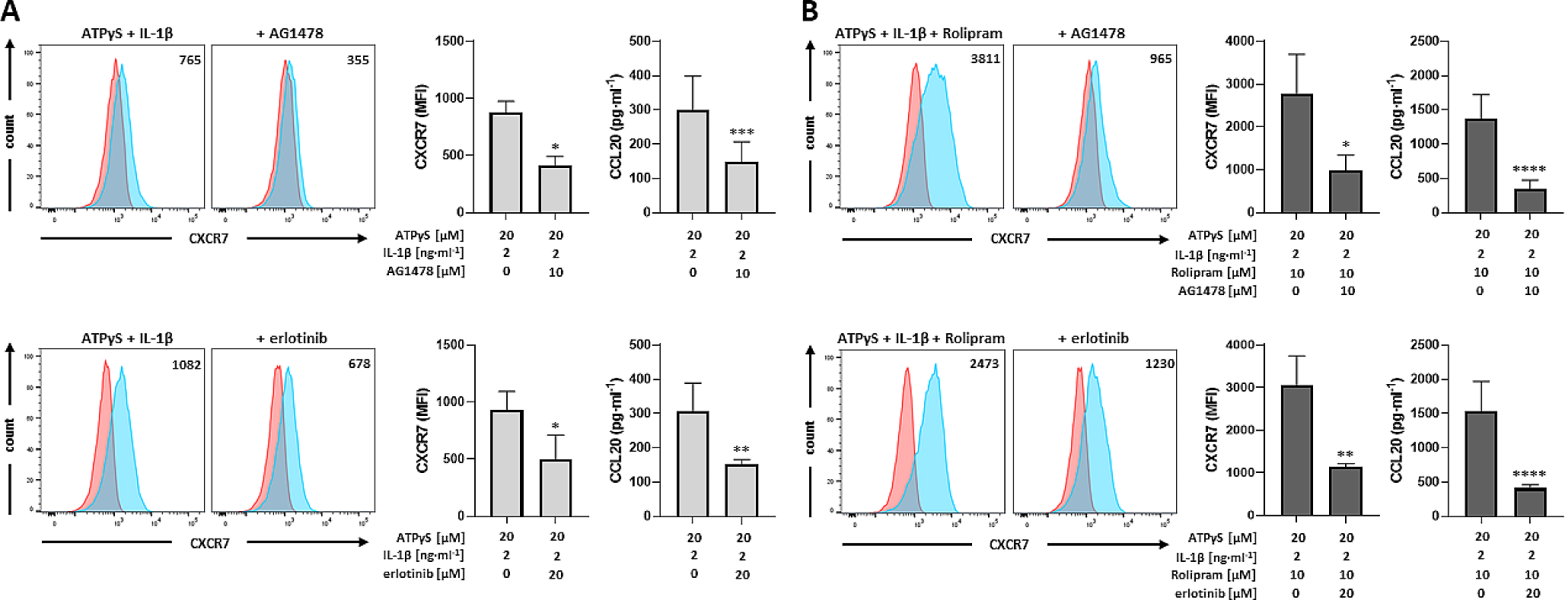
P2Y_11_/IL-1R induced and rolipram-enhanced CXCR7 upregulation in primary human M2 macrophages is mediated by EGFR. **a** M2 macrophages were treated for 24 h with P2Y_11_ receptor agonist ATPγS plus IL-1ß to induce CXCR7 upregulation. (B) Potentiation of CXCR7 expression by PDE4 inhibitor rolipram. **a,b** To examine EGFR involvement, EGFR-TKIs AG1478 and erlotinib were used to modulate P2Y_11_/IL-1R induced and rolipram-enhanced CXCR7 upregulation. Representative FACS histograms of CXCR7 expression (light blue; isotype controls in red) are shown in the left panel. Numbers represent mean fluorescence intensities (MFIs) of the respective staining after subtraction of isotype control MFIs. **a,b** Quantification of CXCR7 expression (*n* = 3) and CCL20 production (*n* = 5) is shown in the right panel. Data shown are mean values ± SD. *p < 0.05, **p < 0.01, ***p < 0.001

This prompted us to examine a potential role of EGFR in P2Y_11_/IL-1R-mediated CXCR7 upregulation. We found that the tyrphostin AG1478 (10 µM), a selective EGFR tyrosine kinase inhibitor (EGFR-TKI), inhibited CXCR7 upregulation induced by P2Y_11_/IL-1R crosstalk with or without PDE4 inhibition (Fig. 2). Erlotinib (Tarceva; 20 µM), another EGFR-TKI, likewise attenuated CXCR7 upregulation. Moreover, inhibition of EGFR-TK activity by either AG1478 or erlotinib inhibited CCL20 production to a similar extent when compared to CXCR7 upregulation (Fig. 2), indicating that EGFR mediates P2Y_11_/IL-1R-induced CXCR7 upregulation and suggesting that an EGFR/CXCR7 axis promotes CCL20 production.

ERK MAP kinase is a downstream effector of both, EGFR and CXCR7 signaling [15, 43]. Consistently, MEK/ERK inhibitor U0126 (10 µM) potently inhibited P2Y_11_/IL-1R-induced and rolipram-enhanced CCL20 production (Fig. S2A).

In our previous studies, we found that P2Y_11_ induced and IL-1R enhanced ADAM17 activity in M2 macrophages, leading to the release (ectodomain shedding) of soluble tumor necrosis factor receptor 2 (sTNF-R2) [30, 31]. ADAM17 may also be responsible for the supply of EGFR ligands such as epiregulin, transforming growth factor α (TGFα), amphiregulin (AREG), and heparin-binding EGF-like growth factor (HB-EGF) [44]. We therefore tested the ADAM17 inhibitor TAPI-1. However, the inhibitory effect of TAPI-1 (20 µM) on P2Y_11_/IL-1R induced CCL20 production was relatively weak and not significant (Fig. S3A). Since ADAM10 might also contribute to the release of EGFR ligands such as EGF and betacellulin (BCT) [44], we used GI 254,023 × (10 µM), which however also failed to attenuate CCL20 production (Fig. S3A).

In search of EGFR ligands, we re-examined a previously performed secretome analysis of P2Y_11_-activated astrocytoma cells [30]. Among the candidates analyzed, only TGFα was secreted in response to P2Y_11_ activation (Fig. S4A). Interestingly, EGFR itself was also released. Ectodomain shedding has been reported to occur in malignant cells that overexpress the EGFR [45]. However, both TGFα and EGFR emerged at much lower levels compared to the known P2Y_11_ target sTNF-RI (Fig. S4A) [30].

We additionally examined the secretome of P2Y_11_/IL-1R activated M2 macrophages for EGFR activators. Amphiregulin, EGF and HB-EGF remained undetectable (Fig. S5A). As in the astrocytoma cells, TGFα was produced in a P2Y_11_-dependent manner in M2 macrophages, although at a low level, contrasting with the high levels of the known P2Y_11_ target sTNF-RII (Fig. S5A) [30].

Based on the secretome data, we focused on antibody-mediated neutralization of TGFα. Despite our failure to detect it, we also included HB-EGF neutralization, because of its involvement in EGFR transactivation in macrophages [46]. However, neither the neutralization of TGFα nor that of HB-EGF could prevent CCL20 production (Fig. S3B). EGFR ectodomain shedding, a feature of malignant cells [45] including astrocytoma cells (Fig. S4A), was not observed in primary human macrophages (Fig. S5A).

Activation of the CXCR7 ligands, CXCL11 (I-TAC) and CXCL12 (SDF-1α) could not be detected in our NanoString-based gene expression analysis (Fig. S5B). In addition, CXCL11 and CXCL12 were low or absent in the astrocytoma (Fig. S4B) and in the M2 macrophage secretome (Fig. S5C). CXCL12 was slightly above the limit of detection (LOD) and sensitive to inhibition with NF340. However, CXCL12 neutralization had no effect on CCL20 production (Fig. S3C), suggesting that CXCR7-mediated EGFR activation in macrophages also occurs in a CXCR7 ligand-independent manner, which is in accordance with observations made in other studies [38, 39].

### CXCR4 is upregulated along with CXCR7 in M2 macrophages: CXCR4 antagonism enhances P2Y_11_/IL-1R-mediated responses

**Fig. 3:**
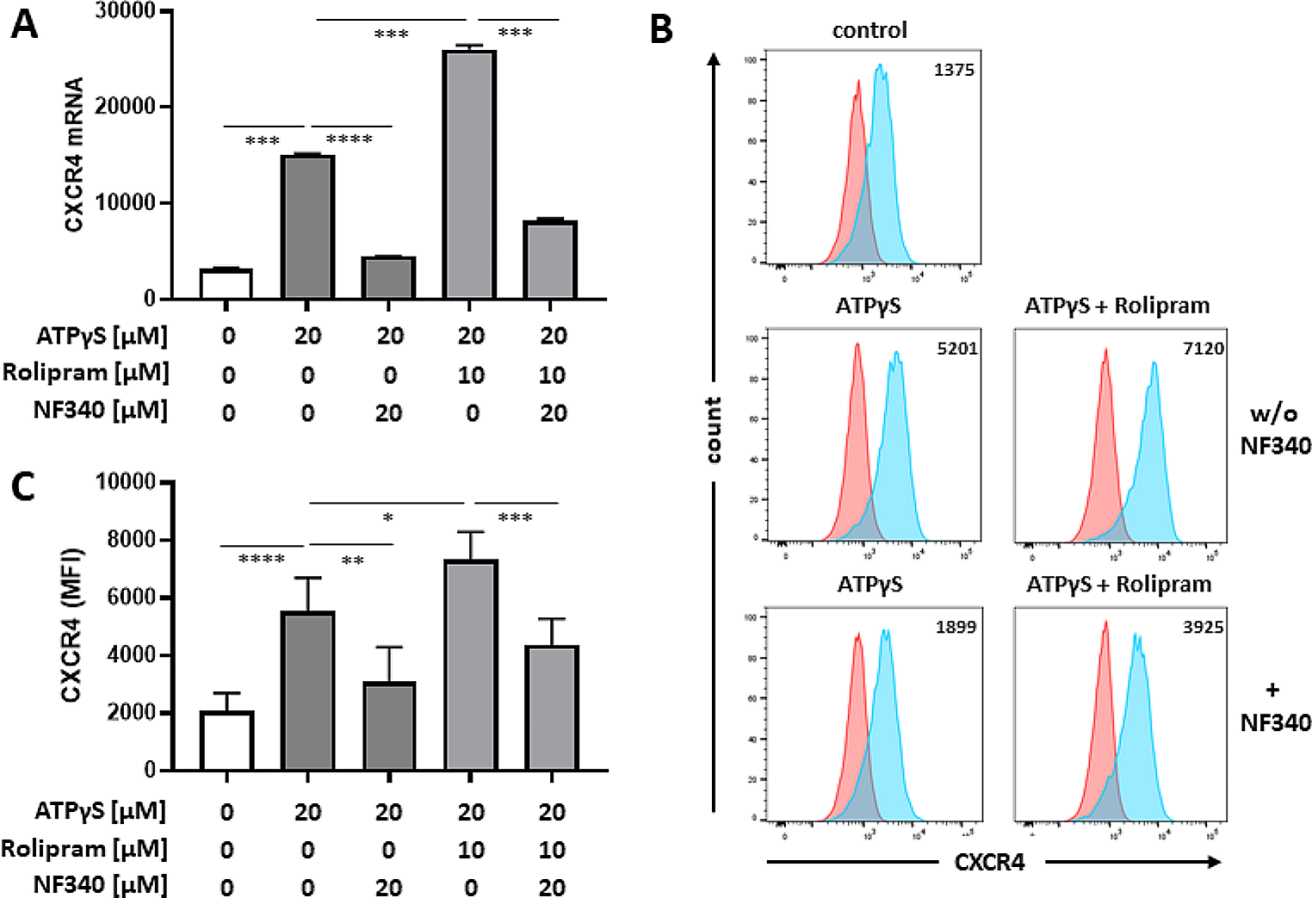
P2Y_11_ activation is sufficient to induce high CXCR4 expression in primary human M2 macrophages: enhancement by PDE4 inhibition with no requirement for IL-1ß co-stimulation. **a** M2 macrophages were treated for 6 h with P2Y_11_ receptor agonist ATPγS ± PDE4 inhibitor rolipram. Copy numbers of CXCR4 mRNA were determined using NanoString technology. P2Y_11_ receptor antagonist NF340 was used to confirm that ATPγS-mediated changes were specific to P2Y_11_ receptor activation. ***p < 0.001, ****p < 0.0001. **b** M2 macrophages were treated for 24 h with P2Y_11_ receptor agonist ATPγS ± PDE4 inhibitor rolipram. Flow cytometry was used to determine CXCR4 expression (light blue; isotype controls in red). Numbers represent mean fluorescence intensities (MFIs) of the respective staining after subtraction of isotype control MFIs. **c** Quantification of CXCR4 expression (*n* = 5). Mean values ± SD are shown. *p < 0.05, **p < 0.01, ***p < 0.001

The second hallmark of P2Y_11_-induced chemokine receptor remodeling was the strong increase of CXCR4 mRNA expression (Table S1). In contrast to CXCR7, IL-1ß was not required for the upregulation of CXCR4 protein. In the absence of IL-1ß, ATPγS alone or in combination with rolipram effectively induced CXCR4 mRNA and protein expression (Fig. 3) at levels well above those of CXCR7 expression (Fig. 1).

To examine CXCR4 function, we tested three different CXCR4 antagonists: (1) the bicyclam AMD070 (X4P-001, mavorixafor), which is a selective CXCR4 antagonist; (2) the bicyclam AMD3100 (plerixafor), which has therapeutic potential in HIV infection and inflammatory diseases including WHIM syndrome as well as in cancer and stem-cell mobilization [47, 48]. In addition to its antagonistic effects at CXCR4, plerixafor may also act as a weak allosteric CXCR7 agonist by promoting ß-arrestin recruitment to CXCR7 [49]; (3) TC14012, a cyclic peptidomimetic inverse agonist of CXCR4 that may also serve as a potent agonist at the CXCR7 [50]. TC14012-mediated activation of a protective CXCR7 pathway has been shown to facilitate tissue repair [20] and ischemia-induced angiogenesis [51].

**Fig. 4:**
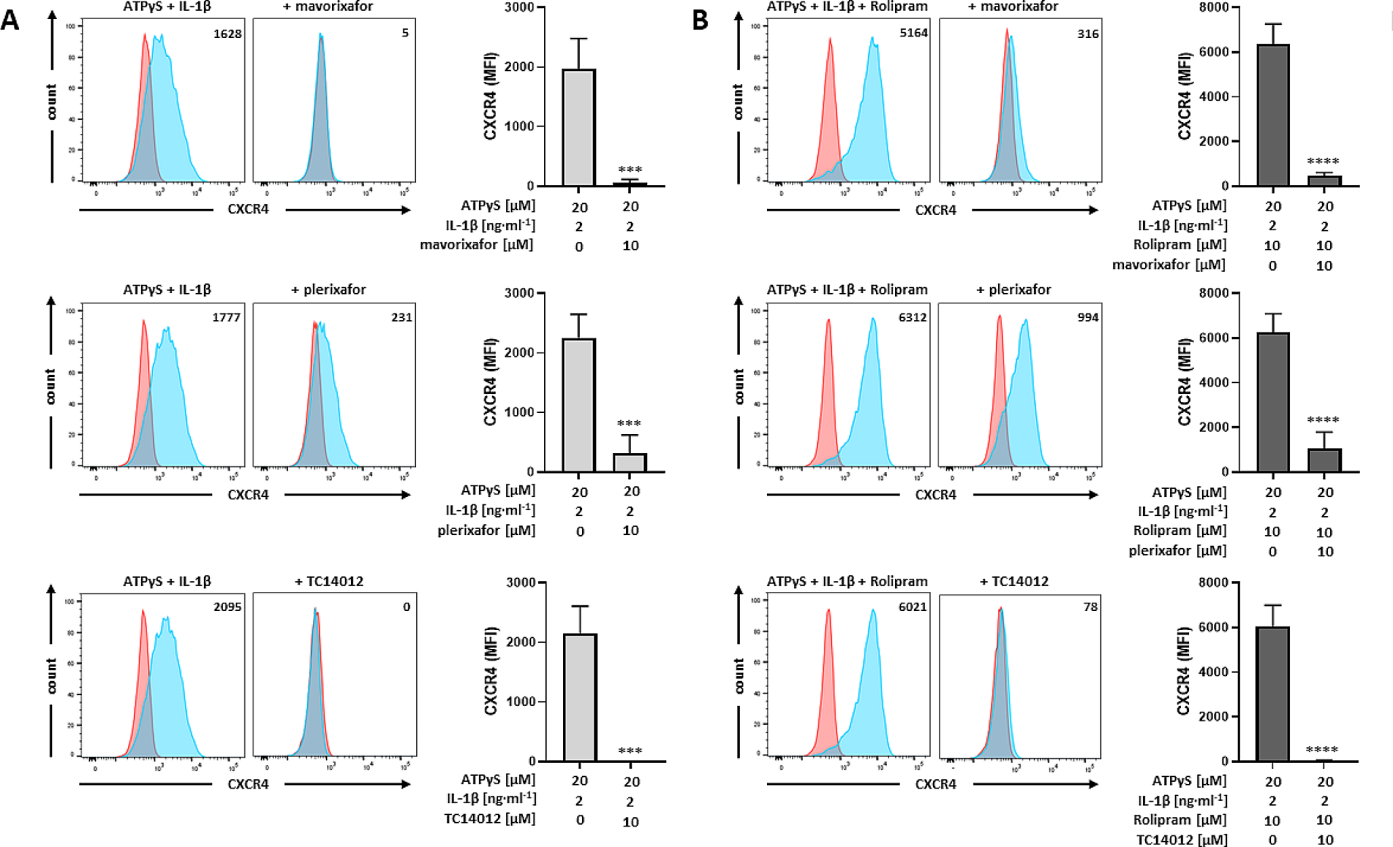
CXCR4 antagonists cause CXCR4 downregulation in primary human M2 macrophages. M2 macrophages were treated for 24 h with P2Y_11_ receptor agonist ATPγS plus IL-1ß in the **a** absence or **b** presence of PDE4 inhibitor rolipram. **a,b** The CXCR4 antagonists mavorixafor, plerixafor and TC14012 were used to modulate P2Y_11_/IL-1R induced and rolipram-enhanced CXCR4 expression. Representative FACS histograms of CXCR4 expression (light blue; isotype controls in red) are shown in the left panel. Numbers represent mean fluorescence intensities (MFIs) of the respective staining after subtraction of isotype control MFIs. **a,b** Quantification of CXCR4 expression is shown in the right panel (*n* = 4). Mean values ± SD are shown. ***p < 0.001, ****p < 0.0001

As a first step, we analyzed the effects of CXCR4 antagonists on CXCR4 expression induced by P2Y_11_/IL-1R activation with or without PDE4 inhibition. All three antagonists (mavorixafor, plerixafor, TC14012), which are known to abrogate CXCR4-induced Gαi signaling [52], were by themselves effective in removing CXCR4 from the cell surface of M2 macrophages (Fig. 4). Consistent with its higher CXCR4 affinity measured in CXCL12 competitive binding assays [53], mavorixafor was also more potent than plerixafor in inducing CXCR4 internalization (Fig. 4).

**Fig. 5:**
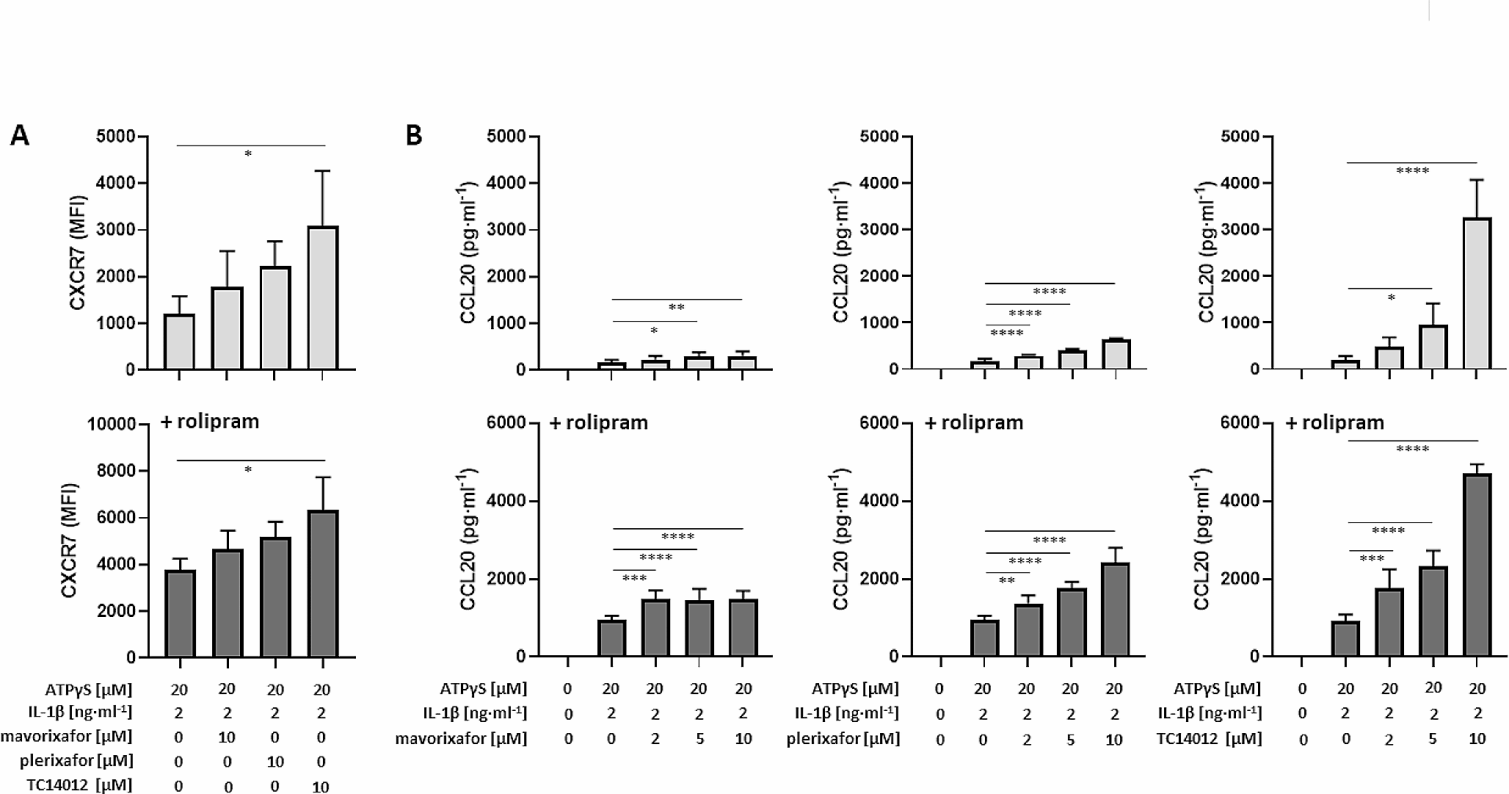
CXCR4 antagonists do not downregulate but rather enhance CXCR7 expression and CCL20 production in primary human M2 macrophages. **a** CXCR7 expression: M2 macrophages were treated for 24 h with P2Y_11_ receptor agonist ATPγS plus IL-1ß in the absence (upper panel) or presence of PDE4 inhibitor rolipram (lower panel). CXCR4 antagonists mavorixafor, plerixafor and TC14012 were used to modulate P2Y_11_/IL-1R induced and rolipram-enhanced CXCR7 expression. Flow cytometry was used to determine CXCR7 expression (*n* = 3; mean fluorescence intensities, MFIs, after subtraction of isotype control MFIs). Mean values ± SD are shown. *p < 0.05. **b** CXCR4 antagonists mavorixafor, plerixafor and TC14012 were used to modulate P2Y_11_/IL-1R induced (upper panels; *n* = 3) and rolipram-enhanced CCL20 production (lower panels; *n* = 3): data shown are mean values ± SD. *p < 0.05, **p < 0.01, ***p < 0.001, ****p < 0.0001

Upon removal from the cell surface, CXCR4 is mostly degraded, which is in contrast to CXCR7 that recycles back to the cell surface [54]. Accordingly, we found that all three antagonists, did not remove CXCR7 from the cell surface (Fig. 5A). Conversely, they even induced a modest increase in CXCR7 expression. Although this was not significant, a slight hierarchy appeared to emerge. While plerixafor was less effective in inducing CXCR4 downregulation, it was more potent in increasing CXCR7 expression, a fact that can be attributed to its additional ability to recruit ß-arrestin2 to CXCR7 [49]. TC14012 was most potent (Fig. 5A) as it can suppress CXCR4 and activate CXCR7 [50], also suggesting that CXCR7 enhances its own expression.

We went on and examined the effects of CXCR4 antagonists on CCL20 production. In accordance with their effects on CXCR7 expression, mavorixafor, plerixafor and TC14012 also enhanced CCL20 production (Fig. 5B). Again, plerixafor was more potent than mavorixafor and TC14012 was most potent. The enhancing effects of all three CXCR4 antagonists were abrogated by MEK/ERK inhibitor U0126 (Fig. S2B). A small molecule ligand known as VUF11207 has also been reported to promote ß-arrestin recruitment to CXCR7 [55] but, surprisingly, VUF11207 did not cause the expected ß-arrestin-facilitated ERK1/2 activation [56]. In accordance with its inability to activate ERK1/2, VUF11207 (100 nM) [57] failed to enhance P2Y_11_/IL-1R initiated CCL20 production (Fig. S2C).

### In glioma cells, naturally expressing CXCR7 but lacking CXCR4, CXCR7 mediates the P2Y_11_/IL-1R driven CCL20 production with no requirement for PDE4 inhibition

**Fig. 6:**
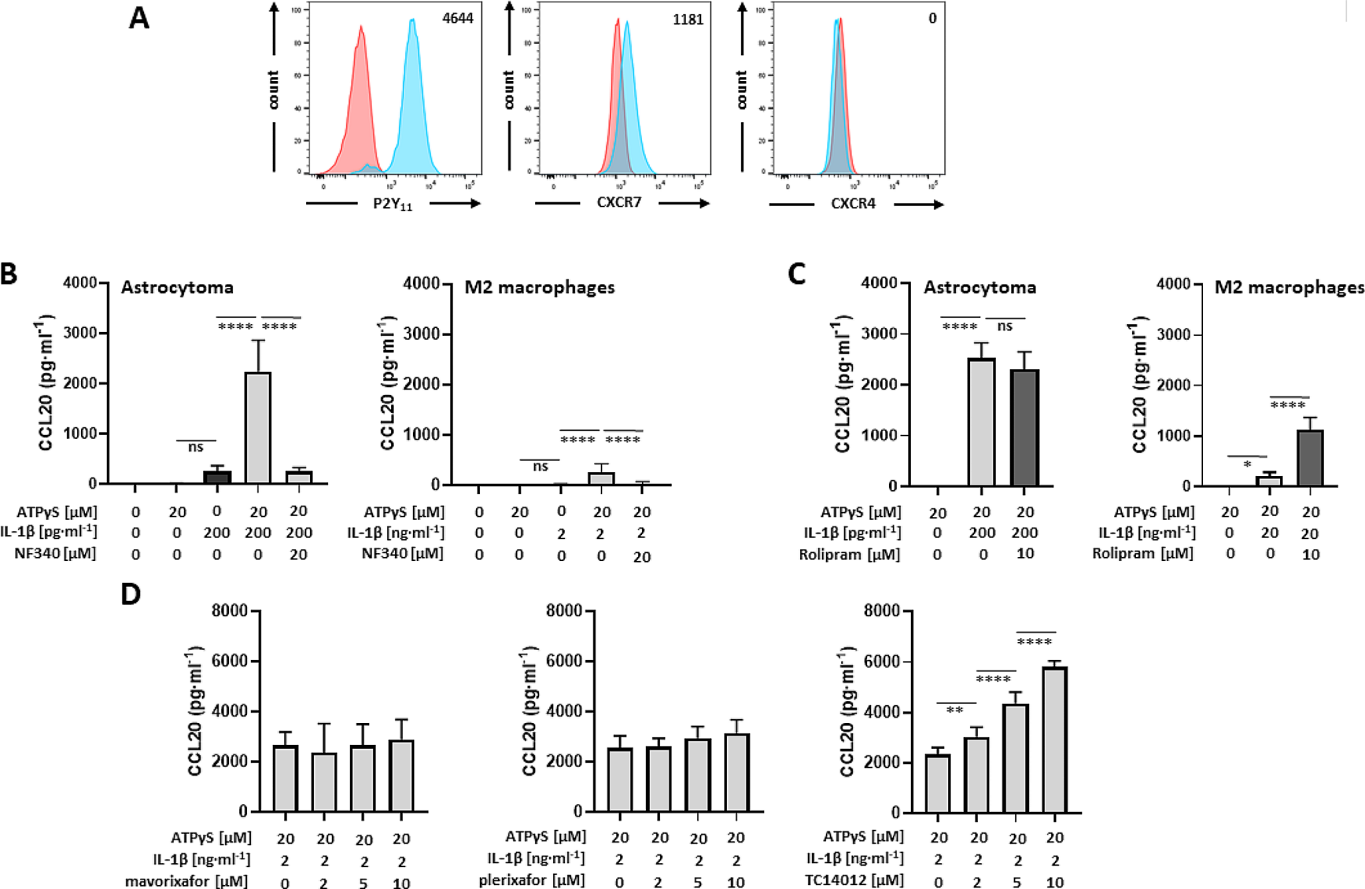
The P2Y_11_-recombinant astrocytoma cell line naturally expresses CXCR7 but lacks CXCR4: enhancement of CCL20 production through CXCR7 activation and no need for PDE4 inhibition. **a** Flow cytometry was used to determine P2Y_11_ receptor as well as CXCR7 and CXCR4 expression (light blue; the respective isotype controls in red). Numbers are mean fluorescence intensities (MFIs) after subtraction of isotype control MFIs. **b** Astrocytoma cells and M2 macrophages were treated for 24 h with P2Y_11_ receptor agonist ATPγS plus IL-1ß in the absence of PDE4 inhibitor rolipram and CCL20 levels were determined (*n* = 5). Mean values ± SD are shown. ****p < 0.0001. **c** Astrocytoma cells and M2 macrophages were treated for 24 h with P2Y_11_ receptor agonist ATPγS plus IL-1ß in the presence of PDE4 inhibitor rolipram and CCL20 levels were determined (*n* = 3). Mean values ± SD are shown. *p < 0.05, ****p < 0.0001. **d** CXCR4 antagonists mavorixafor, plerixafor and TC14012 were used to modulate P2Y_11_/IL-1R induced CCL20 production (*n* = 3). Data shown are mean values ± SD. **p < 0.01, ****p < 0.0001

TC14012 acts both, as a CXCR4 antagonist and as a CXCR7 agonist. To clarify the mechanism of TC14012-enhanced CCL20 production, we took advantage of a P2Y_11_-recombinant astrocytoma cell line, which we already used in our previous studies [27, 30, 31]. Astrocytoma, which are grade II gliomas, often overexpress CXCR7 [21]. High levels of CXCR7 render glioma resistant to drug-induced apoptosis [21]. Importantly, however, most astrocytoma cell lines lack CXCR4 [21]. Accordingly, we were able to detect strong CXCR7 expression in unstimulated P2Y_11_-recombinant astrocytoma cells using flow cytometry (Fig. 6A). In contrast, CXCR4 remained undetectable. This cell system thus represents a sort of natural CXCR4 knockout that allows us to examine P2Y_11_/IL-1R/CXCR7 crosstalk that is unaffected by CXCR4.

**Fig. 7:**
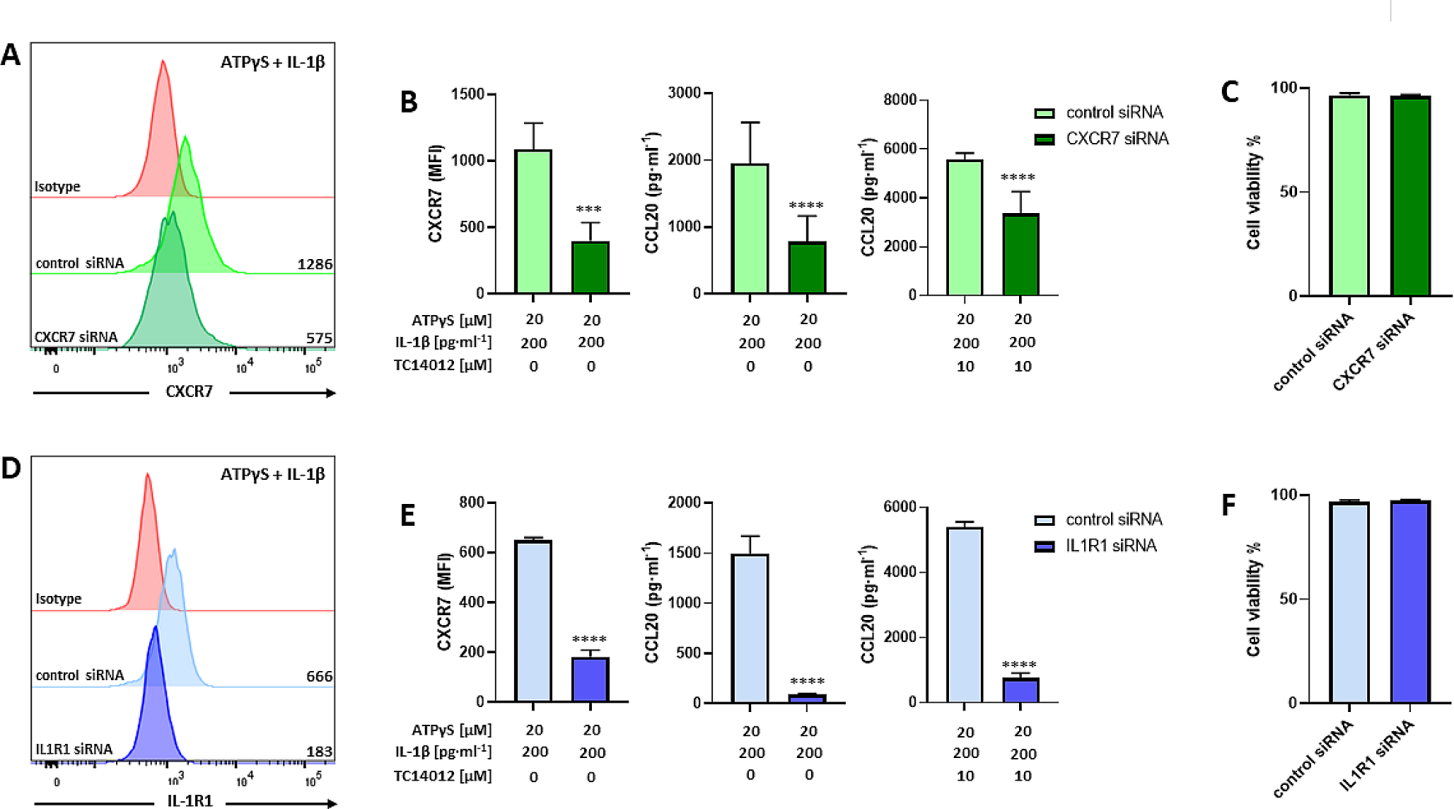
CXCR7 knockdown by RNA interference in astrocytoma cells attenuates CCL20 expression. **a** Astrocytoma cells were transfected with either control siRNA or CXCR7 siRNA for 48 h, with or without subsequent P2Y_11_/IL-1R stimulation for 24 h. The level of knockdown was controlled by measuring CXCR7 surface expression by flow cytometry. Numbers are mean fluorescence intensities (MFIs) after subtraction of isotype control MFIs. **b** Quantification of CXCR7 knockdown (*n* = 4): mean values ± SD are shown. ***p < 0.001. ATPγS/IL-1ß induced and TC14012-enhanced CCL20 production was determined in control cultures (control siRNA) or in cultures subjected to CXCR7 knockdown (CXCR7 siRNA; *n* = 5): mean values ± SD are shown. ****p < 0.0001. **c** Cell viability was determined by eFluor 780-based dead cell exclusion (*n* = 4): mean values ± SD shown. **d** Astrocytoma cells were transfected with either control siRNA or IL1R1 siRNA for 48 h, with or without subsequent P2Y_11_/IL-1R stimulation for 24 h. The level of knockdown was controlled by measuring IL-1R1 expression by flow cytometry. Numbers are mean fluorescence intensities (MFIs) after subtraction of isotype control MFIs. **e** Quantification of IL1-R1 knockdown (*n* = 4): mean values ± SD are shown. ****p < 0.0001. ATPγS/IL-1ß induced and TC14012-enhanced CCL20 production was determined in control cultures (control siRNA) or in cultures subjected to IL1R1 knockdown (IL1R1 siRNA; *n* = 3): mean values ± SD are shown. ****p < 0.0001. **f** Cell viability was determined by eFluor 780-based dead cell exclusion (*n* = 4): mean values ± SD shown

In line with our hypothesis that CXCR4 has regulatory effects, P2Y_11_/IL-1R signaling was sufficient to induce CCL20 production in astrocytoma cells with no requirement for PDE4 inhibition (Fig. 6B). In addition, compared to macrophages, a 10-fold lower dose of IL-1ß (0.2 ng/ml) was sufficient to induce high-level CCL20 production (Fig. 6B). Moreover, PDE4 inhibition did not further increase CCL20 production (Fig. 6C). Consistent with the lack of CXCR4, the selective CXCR4 antagonist mavorixafor failed to enhance CCL20 production (Fig. 6C). Plerixafor was also ineffective. Only TC14012 was able to significantly increase CCL20 production (Fig. 6D). This finding not only established that TC14012 acts as a CXCR7 agonist in our experimental system but also strongly pointed to CXCR7 as a critical signaling component in P2Y_11_/IL-1R driven CCL20 production. To clearly define CXCR7 gene function, we resorted to CXCR7 knockdown experiments using RNA interference. Data shown in Fig. 7 indicate that short interfering RNA (siRNA) attenuated CXCR7 expression (Fig. 7A) as well as P2Y_11_/IL-1R-driven and TC14012-enhanced CCL20 production (Fig. 7B). Of note, CXCR7 knockdown did not impair cell viability (Fig. 7C). For control purposes, we also performed IL1R1 knockdown, which likewise abolished CCL20 production without impairing cell viability (Fig. 7D-F).

### Concomitant activation of P2_11_ and CXCR7 results in CCL20 production with no requirement for IL-1ß stimulation or PDE4 inhibition

**Fig. 8:**
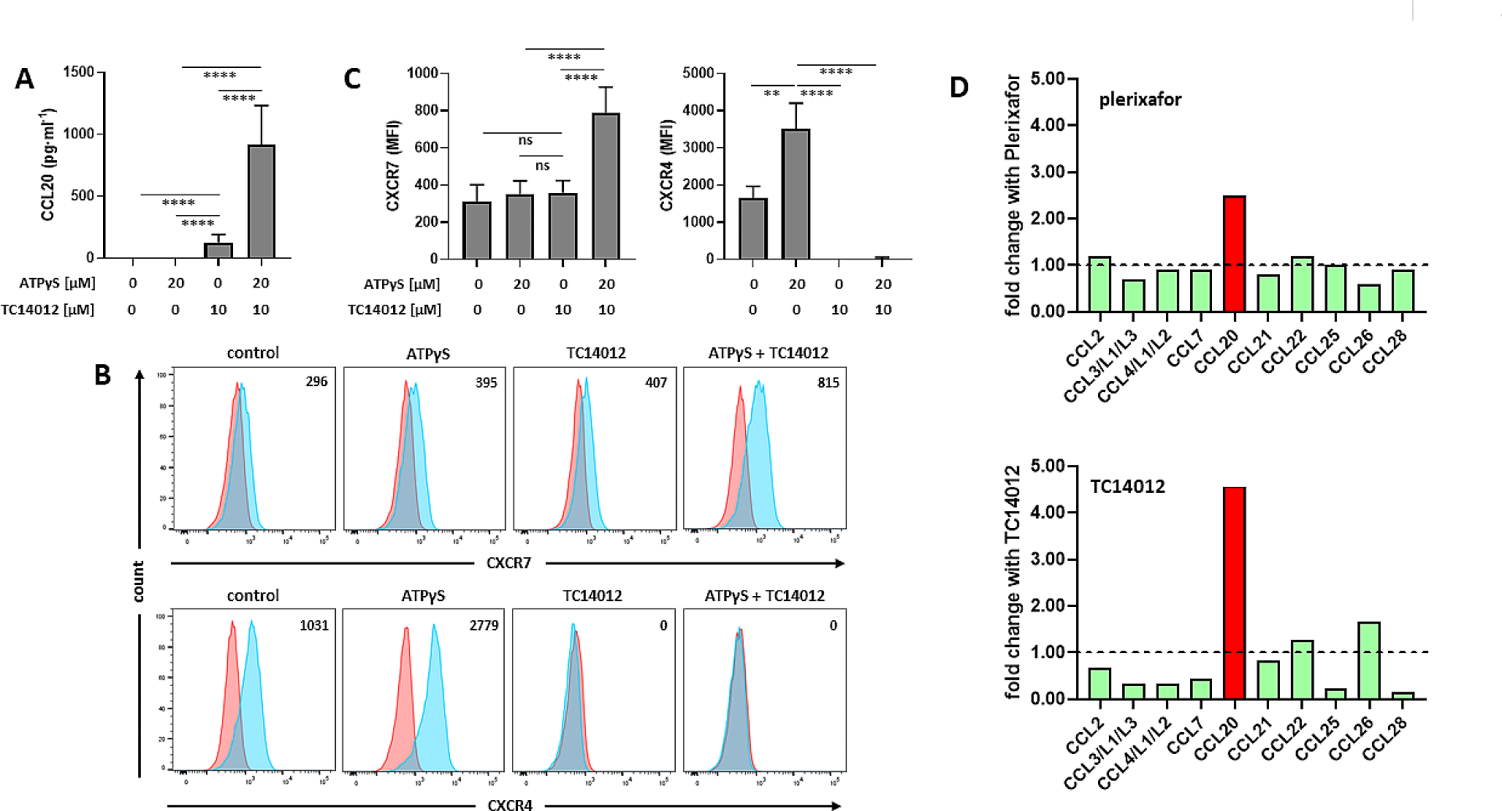
Synergistic cooperation between P2Y_11_ and CXCR7 in primary human macrophages eliminates the need for IL-1ß co-stimulation and PDE4 inhibition. **a** M2 macrophages were treated for 24 h with P2Y_11_ receptor agonist ATPγS and CXCR7 agonist TC14012, alone or in combination, and CCL20 was determined in culture supernatants (*n* = 5). Mean values ± SD are shown. ****p < 0.0001. **b** Flow cytometry was used to determine CXCR7 and CXCR4 expression (light blue; isotype controls in red). Numbers represent mean fluorescence intensities (MFIs) of the respective staining after subtraction of isotype control MFIs. **c** Quantification of CXCR7 (*n* = 5) and CXCR4 expression (*n* = 3). Mean values ± SD are shown. ***p < 0.001, ****p < 0.0001. **d** M2 macrophages were treated for 24 h with P2Y_11_ receptor agonist ATPγS plus IL-1ß in the presence or absence of either CXCR4 antagonist plerixafor or CXCR7 agonist TC14012. Pooled supernatants were analyzed for the presence of 20 CCL chemokines using RayBio technology. Plerixafor- or TC14012-mediated modulation of CCL chemokines that were present in the secretome and produced in a P2Y_11_ receptor dependent manner (i.e. sensitive to inhibition with NF340) is shown (see also Fig. S9)

In M2 macrophages, triggering P2Y_11_ with ATPγS was in itself insufficient and failed to stimulate CCL20 production (Fig. 8A). TC14012-mediated CXCR7 activation caused only weak CCL20 production. In contrast, the concomitant activation of P2Y_11_ and CXCR7 resulted in the upregulation of CCL20 secretion, indicating the synergistic cooperation of the two receptors. Intriguingly, this response occurred in the absence of IL-1ß treatment and PDE4 inhibition with rolipram. Flow cytometric analysis of CXCR4 and CXCR7 regulation revealed that simultaneous activation of P2Y_11_ and CXCR7 increased CXCR7 and abolished CXCR4 surface expression (Fig. 8B/C). Again, P2Y_11_ antagonist NF340 as well as EGFR inhibitors AG1478 and erlotinib prevented the response (Fig. S6). Altogether, the data indicated that concomitant CXCR7 activation rendered P2Y_11_-driven CCL20 production independent of IL-1ß and PDE4 inhibition, and this appears to be due to increased CXCR7 expression and complete CXCR4 downregulation. In accordance with our findings in macrophages, P2Y_11_/IL-1R- or P2Y_11_/CXCR7-driven CCL20 production in astrocytoma cells depended on EGFR as well as on ERK (Fig. S7) and appeared to occur in an EGFR/CXCR7-ligand-independent manner (Fig. S8).

### Analysis of CCL chemokines in the P2Y_11_/IL-1R activated macrophage secretome reveals selective reinforcement of CCL20 secretion in response to TC14012 and plerixafor

In our previous gene expression analysis [31], among 24 CCL chemokines analyzed only CCL20 was upregulated in response to P2Y_11_ activation. To identify additional CCL chemokines induced by P2Y_11_/IL-1R crosstalk and enhanced by CXCR4/CXCR7 manipulation, we performed a secretomic screen based on RayBio technology (Fig. S9A). In the first step, we investigated which CCL chemokines were present in culture medium conditioned by P2Y_11_/IL-1R activated macrophages and identified those that were subject to inhibition with P2Y_11_ antagonist NF340. CCL11, CCL14, CCL15, CCL16, CCL17, CCL19, CCL27 could not be detected. In contrast, CCL1, CCL5, CCL8, CCL13, CCL18, CCL23, CCL24 were present in macrophage-conditioned medium but the secretion of these chemokines was resistant to inhibition with the P2Y_11_ antagonist NF340 (Fig. S9B). Conversely, CCL2, CCL3/L1/L3, CCL4/L1/L2, CCL7, CCL20, CCL21, CCL22, CCL25, CCL26, CCL28 turned out to be subject to inhibition with NF340 and these 10 chemokines are thus likely targets of P2Y_11_/IL-1R crosstalk (Fig. S9C). Besides CCL20, which is well established, CCL21 and CCL4/L1/L2 are prime candidates as P2Y_11_/IL-1R targets. In the second step, we tested whether the remaining 10 chemokines were enhanced by plerixafor and TC14012. To our surprise, CCL20 was the only P2Y_11_/IL-1R induced chemokine that was enhanced by plerixafor and TC14012 (Fig. 8D), indicating that the P2Y_11_/IL-1R/CXCR7 axis stimulates CCL chemokine secretion in a rather selective manner.

## Discussion

**Fig. 9:**
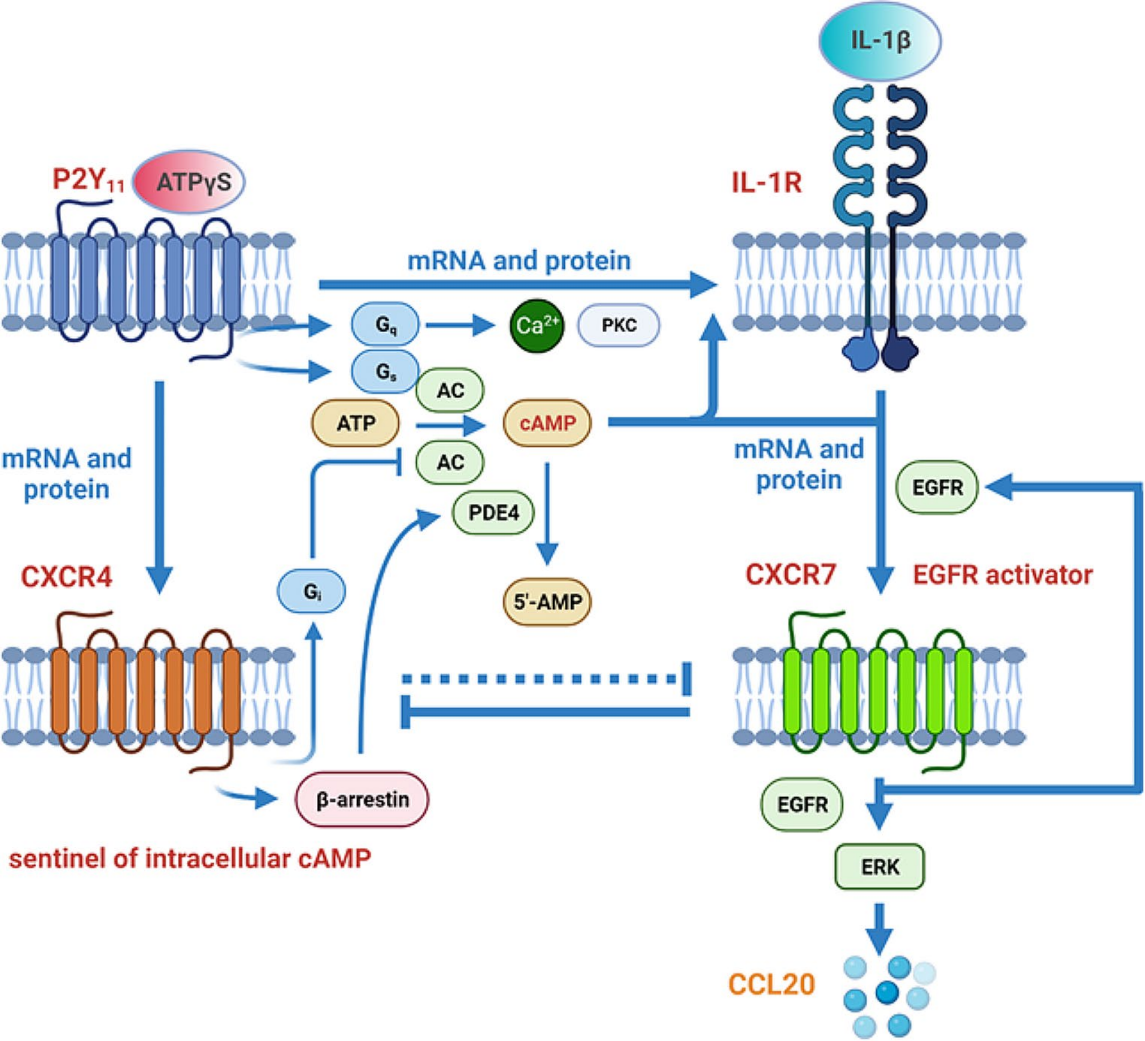
Working model of P2Y_11_ crosstalk with IL-1R and chemokine receptors summarizing current findings and knowledge. P2Y_11_ couples to G_q_ and G_s_ proteins. While G_q_ activates phospholipase Cß and thus initiates the mobilization of Ca^2+^ (via inositol triphosphate, IP_3_) as well as the activation of protein kinase C (PKC, via diacylglycerol, DAG), G_s_ activates adenylyl cyclase (AC) to increase the levels of cyclic adenosine monophosphate (cyclic AMP). P2Y_11_ upregulates CXCR4 and via IL-1R also CXCR7. Upregulation of IL-1R, CXCR7 and CCL20 depends on cyclic AMP. Epidermal growth factor receptor (EGFR) supports upregulation of CXCR7, which serves as an EGFR activator potentiating its signaling capacity, generating a feed-forward loop. Thus, CXCR7 stimulates its own expression with the help of EGFR. By coupling to G_i_, which blocks the cyclic AMP-generating enzyme AC, and by recruiting the cyclic AMP-degrading enzyme PDE4 via ß-arrestin, CXCR4 serves as a perfectly suited sentinel of intracellular cyclic AMP levels and thus as a regulatory checkpoint of P2Y_11_/IL-1R induced and EGFR/CXCR7-mediated responses (dashed inhibitory arrow) such as the selective activation of CCL20 secretion. Once CXCR7 is fully activated by TC14012, it takes control and eliminates CXCR4 expression (inhibitory arrow) and thus also the need for rolipram-mediated PDE4 inhibition

The ATP receptor P2Y_11_ is an unconventional GPCR. It is the only member of the P2Y family that couples to G_q_ and G_s_ proteins (Fig. 9) [24]. P2Y_11_’s other peculiarity is that it cannot be detected in rodents. Although P2Y_11_ has often been examined in immune cells [36, 58, 59], its role in these cells is still far from being fully clarified. Previously, we observed P2Y_11_ upregulation during M-CSF-driven differentiation of monocyte-derived macrophages [27] and crosstalk with the IL-1R [30]. In these studies, we identified chemokines and, in particular, CCL20 as a prime target of the receptor [31].

CCL20 not only supports tissue homeostasis, it is upregulated during inflammatory processes and promotes host defense against pathogens through recruiting CCR6-expressing immune cells including Th17 T cells to maintain barrier immunity in the skin and at mucosal surfaces [60]. The dysregulation of CCL20 or CCR6 can give rise to a large number of diseases including many inflammatory and autoimmune diseases [61]. In Covid-19, severe disease was shown to be associated with inflammatory macrophages producing CCL20 [62]. In addition, the cytokine profile of a novel multisystem inflammatory syndrome in children (MIS-C) caused by severe acute respiratory syndrome coronavirus 2 (SARS-CoV-2) likewise contained CCL20 [63]. The pathogenic role of CCL20-induced CCR6 signaling is particularly well established in cancer. In the tumor microenvironment, CCL20 can cause the exhaustion of CCR6^+^ tumor-infiltrating T cells and concomitantly support the growth and metastasis of CCR6^+^ tumor cells [64].

In the present work, we demonstrate that P2Y_11_ governs chemokine receptor expression and function in human monocyte-derived M2 macrophages. We report 5 major observations: (1) P2Y_11_ selectively activated CXCR4 and CXCR7. (2) P2Y_11_ and IL-1R cooperatively activated EGFR, which in turn upregulated CXCR7 expression. (3) CXCR7 activation enhanced and CXCR7 depletion abolished P2Y_11_/IL-1R-driven CCL20 production. (4) The P2Y_11_/IL-1R-initiated EGFR-CXCR7 crosstalk is controlled by a CXCR4-PDE4 axis. In the presence of CXCR4 (macrophages), effective CCL20 production depended on CXCR4 suppression or PDE4 inhibition with rolipram. Absence of CXCR4 expression (astrocytoma) eliminated the need of PDE4 inhibition, altogether suggesting that CXCR4 serves as a checkpoint that regulates CXCR7 effects by keeping intracellular cyclic AMP levels low. (5) Among the members of the CCL chemokine family, CCL20 is selectively secreted in response to P2Y_11_/IL-1R induced EGFR/CXCR7 crosstalk.

The ability of P2Y_11_ to control chemokine receptor expression emerged from a NanoString-based gene expression analysis, comprising 20 chemokine receptor genes. P2Y_11_ activation led to the highly selective upregulation of the genes encoding CXCR4 and CXCR7. In contrast to CXCR4, which was readily upregulated by P2Y_11_ agonist alone, P2Y_11_-driven CXCR7 mRNA and protein expression strongly depended on PDE4 inhibition with rolipram and on IL-1R stimulation with IL-1ß. The requirements for robust CXCR7 expression are obviously the same as those for CCL20 production [31], implicating CXCR7 in the P2Y_11_/IL-1R-initiated cascade leading to CCL20 production. Given the need for elevated cyclic AMP levels in P2Y_11_/IL-1R-driven CXCR7 expression and CCL20 production, CXCR4, with its known ability to control intracellular cyclic AMP levels via G_i_-mediated AC inhibition [11] as well as by ß-arrestin-mediated PDE4 scaffolding [12], is likely to be the regulatory checkpoint in this system (Fig. 9).

The parallel use of two distinct P2Y_11_-expressing cellular models was very helpful in our subsequent attempts to delineate both, a CCL20-stimulating P2Y_11_/IL-1R/EGFR/CXCR7 axis as well as a CCL20-suppressing CXCR4/PDE4 axis. We used primary human macrophages naturally expressing P2Y_11_, CXCR4 and CXCR7 on the one hand, and on the other, a P2Y_11_-recombinant astrocytoma cell line that naturally expresses CXCR7 but lacks CXCR4. In our side-by-side examinations, the astrocytoma cell line thus served as a natural CXCR4-knockout.

TC14012, initially identified as a peptidomimetic inverse agonist of CXCR4, was later found to additionally act as a CXCR7 agonist [50]. In astrocytoma cells, the CCL20-enhancing effect of TC14012 can only be mediated by CXCR7, as these cells lack CXCR4. The critical role of CXCR7 in the P2Y_11_/IL-1R elicited cascade leading to CCL20 production was confirmed by CXCR7 knockdown, which clearly attenuated CCL20 production. Finally yet importantly, the absence of CXCR4 in astrocytoma cells eliminated the need for PDE4 inhibition. Moreover, a 10-fold lower IL-1ß concentration was sufficient to induce high-level CCL20 production. Astrocytoma, which are grade II gliomas, upregulate CXCR7 to acquire resistance against drug-induced apoptosis [21]. In astrocytoma, tumor progression may not be compatible with CXCR4 expression, since, according to our observations, CXCR4 would limit the pro-survival effect of CXCR7.

The chronic suppression of intracellular cyclic AMP through a CXCR4/PDE4 axis in M2 macrophages explains the need for rolipram and IL-1ß in P2Y_11_-driven CCL20 production. PDE4 inhibition allows the increase of cyclic AMP, which facilitates IL-1R upregulation [30, 31] and, thus, the subsequent IL-1ß driven CXCR7 mRNA and protein expression [14]. IL-1ß not only serves as a second signal for its own expression [35] but also for the expression of CCL20 [31] and obviously also for CXCR7. Concomitant activation of P2Y_11_ and CXCR7 revealed a synergistic cooperation of the receptors, which was accompanied by complete CXCR4 downregulation and thus independent of IL-1ß and rolipram. Our gene expression analysis revealed that P2Y_11_ activation is sufficient to induce CXCR7 mRNA expression, especially when PDE4 is inhibited. Concomitant CXCR7 activation by TC14012 eliminated CXCR4 expression and thus the need of PDE4 inhibition. The fact that IL-1ß was no more required for CCL20 production suggested that CXCR7, similar to IL-1ß [35], supports its own mRNA translation. Similar observations of reciprocal regulation of CXCR4 and CXCR7 have been made in prostate cancer cells [39] and in neurons [65].

The use of macrophages was prerequisite to the observation that EGFR may also act upstream of CXCR7 in primary cells. In transformed cells, CXCR7 is considered an EGFR activator [38, 39]. Constitutive expression of both receptors is a feature of certain types of malignancies such as prostate and breast cancer. In these studies, CXCR7 depletion attenuated EGFR activation [38, 39], thus establishing a signaling cascade in which CXCR7 activation precedes that of EGFR. In contrast, in our studies with primary macrophages, EGFR was responsible for CXCR7 upregulation. The observations made in primary and in transformed cells collectively suggest that EGFR upregulates CXCR7 as an accessory EGFR-activating receptor to potentiate its own signaling (Fig. 9). In accordance with observations made in breast and prostate cancer cells [38, 39], we found that CXCR7-mediated EGFR activation also appears to be ligand-independent in primary human macrophages.

The comparison of primary macrophages with transformed astrocytoma cells has limitations. However, in both systems P2Y_11_/IL-1R activation led to CCL20 production. Moreover, in either cell type inhibition of EGFR (erlotinib, AG1478) and MEK/ERK (U0126) reduced the levels of CCL20, while CXCR7 activation with TC14012 enhanced CCL20 production. In the astrocytoma cell line CXCR7 knockdown diminished CCL20 production. Altogether, these findings suggested that the signaling pathways in the two cellular models are very similar. Consistent with the absence of CXCR4 and the resulting lack of requirement for PDE4 inhibition, a ten-fold lower concentration of IL-1ß was sufficient to induce high levels of CCL20 in the astrocytoma cells. This is not surprising, because in the absence of PDE4 activity, P2Y_11_ can effectively activate cyclic AMP signaling, which is required for the upregulation of IL-1R [30, 31].

Concerned with the question of why CCL20 is induced so selectively in our studies, we found that CCL20 has also been outstanding in other studies. CCL20 was one of the most strongly activated genes in response to p53 activation in human monocyte-derived macrophages [66] or upon ligation of Fas (CD95) in HeLa cells [67]. Moreover, CCL20 production appears to be closely linked to conditions of lipid overload [68]. Altogether, these observations make CCL20 appear to be a critical component of a distinct stress response. There are good reasons to suggest that this stress response is very particular to humans. P2Y_11_ itself is a human-specific receptor inasmuch as it does not occur in mice. Moreover, in side-by-side examinations of human and mouse macrophages, CCL20 specifically emerged as a human-specific target of Toll-like receptor 4 (TLR4) [69]. Of note, TLR4 shares the MyD88-dependent pathway with IL-1R [70]. Collectively, these observations point to a human-specific cytoprotective signaling pathway that is initiated by P2Y_11_/IL-1R crosstalk and leads to CCL20 production. The effects of P2Y_11_ including its downstream product CCL20 would therefore escape observation, when only rodent models are used in basic research and in preclinical drug development, thus contributing to the known gap between animal trials and clinical outcomes.

In summary, we provide evidence that the G protein-coupled ATP receptor P2Y_11_ supports via EGFR the selective upregulation of the chemokine receptor CXCR7 in human macrophages. CXCR7 expression as well as its ability to stimulate CCL20 production depend on elevated levels of intracellular cyclic AMP. CXCR4 controls CXCR7 expression and activity through PDE4-mediated cyclic AMP degradation. Once fully activated, CXCR7 takes control and suppresses CXCR4, facilitating unrestrained CXCR7-mediated responses. Given the cytoprotective potential of P2Y_11_/CXCR7 signaling that includes anti-inflammatory and proangiogenic effects, it is obvious that a lack of it may facilitate the development of inflammatory and autoimmune diseases. Conversely, cancer cells may exploit such mechanisms to survive, progress and develop drug resistance.

## Materials and methods

### Reagents

The ATP analog ATPγS (Sigma Aldrich, St. Louis, MO, USA) and the suramin analog NF340 (Santa Cruz, Dallas, TX, USA) were utilized as P2Y_11_ receptor agonist (20 µM) and antagonist (20 µM), respectively. Additional reagents employed in this study: the PDE4-selective inhibitor rolipram (10 µM) (Sigma-Aldrich), recombinant IL-1β (0.2 and 2 ng·ml^− 1^) (R&D Systems, Minneapolis, MN, USA), the selective EGFR tyrosine kinase inhibitors AG1478 (10 µM) (Sigma) and erlotinib (20 µM) (Cayman Chemical, Ann Arbor, MI, USA), the CXCR4 chemokine receptor antagonists mavorixafor (AMD 070; 2–10 µM), plerixafor (AMD 3100; 2–10 µM), which is also an allosteric agonist of CXCR7, and TC14012 (2–10 µM), which is also a potent CXCR7 agonist (all from MedChemExpress, Monmouth Junction, NJ, USA), the MEK/ERK inhibitor U0126 (10 µM) (Sigma), the ACKR3 (CXCR7) chemokine receptor agonist VUF11207 (100 nM) (MedChemExpress), the TACE/ADAM17 inhibitor TAPI-1 (20 µM) and the ADAM10 inhibitor GI 254,023 × (10 µM) (both from Tocris, Bristol, UK), a polyclonal goat anti-human HB-EGF antibody (10 µg·ml^− 1^) (AF-259-SP; R&D Systems, Minneapolis, MN, USA), a polyclonal goat anti-human TGFα antibody (10 µg·ml^− 1^) (AB-239-NA; R&D Systems), a polyclonal goat anti-human CXCL12 antibody (10 µg·ml^− 1^) (PA1-20154; Invitrogen / Thermo Fisher Scientific, Waltham, MA, USA), and a monoclonal mouse anti-human CXCL11/I-TAC antibody (10 µg·ml^− 1^) (MAB672; R&D Systems).

### Monocyte isolation and generation of M2 macrophages

Buffy coats from randomly selected healthy volunteer donors were provided anonymously by the Central Institute for Blood Transfusion (Medical University of Innsbruck, Austria) after written informed consent. Thus, we have no access to data on age or sex of the blood donors incorporated in this work. Inclusion of healthy donors in the present study was approved by the local institutional review board (ethics committee number: 1087/2018) according to the tenets of the Helsinki Protocol.

Isolation of PBMCs was achieved by density gradient centrifugation (Lymphoprep; Stem Cell Technologies, Vancouver, Canada). Next, monocytes were isolated from PBMCs by positive selection using CD14 microbeads (human; 130-050-201, Miltenyi Biotec, Bergisch Gladbach, Germany) and LS columns (130-042-401, Miltenyi Biotec). Within 6 days, freshly isolated monocytes were differentiated toward M2 macrophages in the presence of M-CSF as described previously [30, 31]. This protocol effectively and reliably generates CD14^high^ macrophage populations that co-express CD163 (haptoglobin-hemoglobin scavenger receptor), which is an M-CSF target gene [71], and CD206 (C-type mannose receptor 1) (Fig. S1) [72]. However, most importantly, these M2-like macrophages express high levels of P2Y_11_ receptors and thus serve as a well-defined cell system for the examination of native P2Y_11_ receptors.

### M2 macrophage stimulation

Fully differentiated M2 macrophages were harvested, washed, and 5 × 10^4^ cells were seeded in 100 µl RPMI1640 (PAN-Biotech, Aidenbach, Germany) supplemented with 5% FBS (HyClone, Logan, UT, USA), 1% GlutaMAX (100x; Gibco / Thermo Fisher Scientific, Waltham, MA, USA), 10 mM HEPES, 1 mM sodium pyruvate (both from PAN-Biotech), 1% NEAA (100x; Gibco), and 1% Pen/Strep ( ≙ 100 units·ml^− 1^ of penicillin, and 100 µg·ml^− 1^ of streptomycin; Gibco) in 96-well plates (Corning/Falcon, New York, USA). For flow cytometry experiments, 1.5 × 10^5^ cells were seeded in 300 µl RPMI1640 (supplemented with the same reagents as described above) in 48-well plates (Corning/Costar, New York, USA). Cells were stimulated in duplicates with the P2Y_11_ receptor agonist ATPγS (20 µM) in the presence or absence of antagonists/inhibitors and/or recombinant cytokines and/or neutralizing antibodies for 24 h at 37 °C in a humidified 5% CO_2_ atmosphere. Supernatants were harvested and cryopreserved at -80 °C.

### Ectopic P2Y_11_ expression

The P2Y_11_-recombinant astrocytoma cell line ES-293-A (P2RY11 cells; purchased from Perkin Elmer, Waltham, MA, USA), which is naturally devoid of functional P2 receptors, has been transfected with human P2RY11 as described previously [30]. The cell line was maintained at 37 °C in a humidified 5% CO_2_ atmosphere in Advanced DMEM (Gibco) supplemented with 10% (v/v) FBS (HyClone), 1 mM sodium pyruvate (PAN-Biotech), 1% GlutaMAX (100x; Gibco), 1% Pen/Strep ( ≙ 100 units·ml^− 1^ of penicillin, and 100 µg·ml^− 1^ of streptomycin; Gibco), and 500 µg·ml^− 1^ of G418 (PAN-Biotech) to select for stable transfectants. One day prior to stimulation, cells were harvested, washed, and 1 × 10^5^ cells were seeded in 400 µl Advanced DMEM supplemented with 10% (v/v) FBS, 1 mM sodium pyruvate, 1% GlutaMAX (100x), 1% Pen/Strep ( ≙ 100 units·ml^− 1^ of penicillin, and 100 µg·ml^− 1^ of streptomycin), and 500 µg·ml^− 1^ of G418. After incubation for 24 h, medium was replaced by 400 µl Advanced DMEM supplemented with 1% (v/v) FBS, 1 mM sodium pyruvate, 1% GlutaMAX (100x), 1% Pen/Strep ( ≙ 100 units·ml^− 1^ of penicillin, and 100 µg·ml^− 1^ of streptomycin), without G418. On the same day, cells were stimulated in duplicates with the P2Y_11_ receptor agonist ATPγS (20 µM) in the presence or absence of antagonists/inhibitors and/or recombinant cytokines and/or neutralizing antibodies for 24 h at 37 °C in a humidified 5% CO_2_ atmosphere. Supernatants were harvested and cryopreserved at -80 °C.

### siRNA-mediated ACKR3/CXCR7 and IL1R1 gene knockdown

ACKR3/CXCR7 or IL1R1 gene knockdown in the P2Y_11_-recombinant astrocytoma cell line (P2RY11 cells) was accomplished by RNA interference employing the ON-TARGETplus SMARTpools targeting ACKR3/CXCR7 or IL1R1. The ON-TARGETplus SMARTpool for ACKR3/CXCR7 (Dharmacon, Horizon Discovery, L-013212-00-0005) contains four different siRNAs targeting ACKR3/CXCR7 (GCCGUUCCCUUCUCCAUUA, UACACGCUCUCCUUCAUUU, GAGCUGGUCUCCGUUGUCU, GCUCAUCGAUGCCUCCAGA). Similarly, the ON-TARGETplus SMARTpool for IL1R1 contains four different siRNAs targeting IL1R1 (GAACACAAAGGCACUAUAA, GCAAAUAGCCAUGUAUAAU, CAUCACAGUGCUUAAUAUA, GGACUUGUGUGCCCUUAUA). The ON-TARGETplus non-targeting control pool (Dharmacon, D-001810-10-05) containing four different siRNAs with no significant homology to any human mRNA (UGGUUUACAUGUCGACUAA, UGGUUUACAUGUUGUGUGA, UGGUUUACAUGUUUUCUGA, UGGUUUACAUGUUUUCCUA) served as negative control. Transfection of P2RY11 cells with respective siRNAs was achieved using Lipofectamine RNAiMAX transfection reagent (Invitrogen) according to the manufacturer’s instructions. Briefly, P2RY11 cells were seeded at 4 × 10^4^ cells per well in a 48-well plate (Corning / Costar) in Advanced DMEM supplemented with 10% (v/v) FBS, 1 mM sodium pyruvate, 1% GlutaMAX (100x), 1% Pen/Strep ( ≙ 100 units·ml^− 1^ of penicillin, and 100 µg·ml^− 1^ of streptomycin), and 500 µg·ml^− 1^ of G418, 24 h prior to transfection. The following day, medium was replaced by 200 µL transfection medium (Advanced DMEM supplemented with 10% (v/v) FBS, 1 mM sodium pyruvate, 1% GlutaMAX without antibiotics) when cells were approximately 40–50% confluent. In order to generate siRNA-lipid complexes, siRNA was diluted in serum-free OptiMEM (Invitrogen) to a final concentration of 50 nM and mixed with diluted Lipofectamine RNAiMAX reagent (1 µL / transfection) in a 1:1 ratio. After 15 min of incubation, P2RY11 cells were forward transfected in duplicates by dropwise addition of 40 µL siRNA-lipid complexes to each well. On the next day, medium was replaced by 400 µL full growth medium to avoid cytotoxic effects of the transfection reagent. In addition, transfection efficiency was controlled after 24 h using the siGLO RISC-Free Transfection Indicator (Dharmacon, D-001630-01-05). Transfection efficiency was > 80%. The level of knockdown was assessed at the protein level 72 h post transfection via quantification of ACKR3/CXCR7 or IL-1R1 expression by flow cytometry.

### Flow cytometry

Staining of cell surface or intracellular antigens was achieved by the use of fluorochrome-conjugated monoclonal (mouse) or polyclonal (rabbit, goat) antibodies. Respective isotype controls were tested in parallel using the same concentration to rule out unspecific background signals. Initially, cells were harvested, washed, and stained with fixable viability dye eFluor 780 (eBioscience / Thermo Fisher Scientific) to exclude dead cells. After another washing step, cells were stained for 30 min at 4 °C in the dark in PBS (Lonza, Basel, Switzerland) containing 0.5% FBS (HyClone) and 50 µg·ml^− 1^ human IgG (Octapharma, Lachen, Switzerland) to block non-specific FC-receptor antibody binding. For intracellular antigen staining, harvested cells were fixed with intracellular fixation buffer (Invitrogen / Thermo Fisher Scientific) for 30 min at RT in the dark after dead cell staining. Following two washing steps, cells were stained in permeabilization buffer (Nordic-MUbio, Susteren, Netherlands) containing 50 µg·ml^− 1^ human IgG for 30 min at RT in the dark. The following antibodies were used: rabbit polyclonal IgG anti-human P2Y_11_ receptor (bs-12071R-A-488; Bioss, Woburn, MA, USA), mouse monoclonal IgG_1_ anti-human P2Y_11_/P2RY11 (FAB9305R-025-AF647; R&D Systems), mouse monoclonal IgG2b anti-human CD14 (clone MϕP9, 345,787-APC; BD Biosciences, Franklin Lakes, NJ, USA), mouse monoclonal IgG1,k anti-human CD163 (clone GHI/61, 556,018-PE; BD Biosciences), mouse monoclonal IgG_1_,k anti-human CD206 (clone 19.2, 17-2069-42-APC, Invitrogen / Thermo Fisher Scientific), mouse monoclonal IgG2a,k anti-human CD184/CXCR4 (clone 12G5, 555,974-PE; BD Biosciences), mouse monoclonal IgG_1_ anti-human ACKR3/CXCR7 (LS-C128442-APC; Lifespan Biosciences, Lynnwood, WA, USA), and goat polyclonal IgG anti-human IL-1R1 (FAB269P-PE; R&D Systems).

All analyses were performed on a FACSCanto II flow cytometer and FACS Diva 6.1.2 as well as FlowJo V7.2.5 software (BD Biosciences) by applying dead cell and doublet discrimination.

### Transcriptome analysis

Total RNA was isolated from fully differentiated M2 macrophages stimulated for 6 h with the P2Y_11_ receptor agonist ATPγS (20 µM) in the presence or absence of the P2Y_11_ receptor antagonist NF340 (20 µM) and/or the PDE4-selective inhibitor rolipram (10 µM). Of each sample, 50 ng of total RNA was used for hybridization reaction with the nCounter Host Response Panel Kit (human; NanoString Technologies, Seattle, WA USA) as described previously [31]. Samples were processed at the Core Facility Molecular Biology at the Centre of Medical Research at the Medical University of Graz. Raw data pre-processing and normalization was conducted as mentioned previously [31]. By using a set of negative control probes, the general gene expression threshold was determined to be 24.7. For statistical analysis, ordinary one-way analysis of variance (ANOVA) was performed and genes with *p* < 0.05 and a fold change of at least 1.5 were considered as differentially regulated.

### Secretome analysis

Fully differentiated M2 macrophages derived from three different donors were stimulated for 24 h with the P2Y_11_ receptor agonist ATPγS (20 µM) plus IL-1β (2 ng·ml^− 1^) with or without the P2Y_11_ receptor antagonist NF340 (20 µM), in the presence or absence of either the CXCR4 antagonist plerixafor (10 µM) or the CXCR4 antagonist and CXCR7 agonist TC14012 (10 µM). As an internal control, cell supernatants of all three donors were analyzed for CCL20 levels to verify the successful stimulation or inhibition of the P2Y_11_/IL-1R axis in the presence or absence of plerixafor or TC14012 (Fig. S9). Pooled supernatants were then sent to a commercial proteomics service (RayBiotech, Peachtree Corners, GA, USA), verified by quality control testing, and analyzed in quadruplicates with the Quantibody Human Cytokine Array Q440 (QAH-CAA-440-1; RayBiotech), a quantitative multiplex ELISA array.

### Cytokine measurements

Levels of CCL20 (MIP-3α) in cell culture supernatants were assessed by ELISA using the Human CCL20/MIP-3 alpha DUO Set ELISA (R&D Systems) according to the manufacturer’s instructions. Evaluations were conducted on an Elx800 universal microplate reader (BioTek Instruments/Agilent, Winooski, VT, USA) coupled to the Gen5 3.09 data analysis software (BioTek Instruments/Agilent).

### Data and statistical analysis

Data are presented as mean values ± SD. Sample sizes and experimental replicates are indicated in Fig. legends. Statistical analyses were performed with the GraphPad Prism software (version 9.4.1). Statistical significance between groups was determined by two-tailed unpaired *t* test. For three or more groups, ordinary one-way analysis of variance (ANOVA) was used, followed by Šídák’s *post-hoc* test. An output of *p* < 0.05 was accepted as significantly different in all tests. Significance levels are: *p < 0.05; **p < 0.01; ***p < 0.001; ****p < 0.0001.

### Electronic supplementary material

Below is the link to the electronic supplementary material.

Supplementary Material 1

## Data Availability

All data generated or analyzed during this study are included in this published article. Additional transcriptome profiling data are also available on request from the corresponding author [MT]. Some data may not be made available because of privacy or ethical restrictions.

## Abbreviations

AC: Adenylyl cyclase
ADAM: A disintegrin and metalloprotease
AMP: Adenosine monophosphate
AREG: Amphiregulin
ATP: Adenosine triphosphate
CCL20: C-C Motif Chemokine Ligand 20
CCR: CC chemokine receptor
CXCL: Chemokine (C-X-C motif) ligand
CXCR: C-X-C motif chemokine receptor
EGFR: Epidermal growth factor receptor
GPCR: G protein-coupled receptor
HB-EGF: Heparin-binding EGF-like growth factor
IL-1R: Interleukin-1 receptor
M-CSF: Macrophage-colony stimulating factor
MIS-C: Multisystem inflammatory syndrome in children
PDE: Phosphodiesterase
TGF: Transforming growth factor
TNFR: Tumor necrosis factor receptor
