# Supplementary material for: Crosstalk between purinergic receptor P2Y11 and chemokine receptor CXCR7 is regulated by CXCR4 in human macrophages
Source: Cell Mol Life Sci. 2024 Mar 13;81(1):132. doi: 10.1007/s00018-024-05158-7 (PMC10933201; doi:10.1007/s00018-024-05158-7)
Supplement: Supplementary file 1 — Supplementary Material 1 [file 18_2024_5158_MOESM1_ESM.pdf]

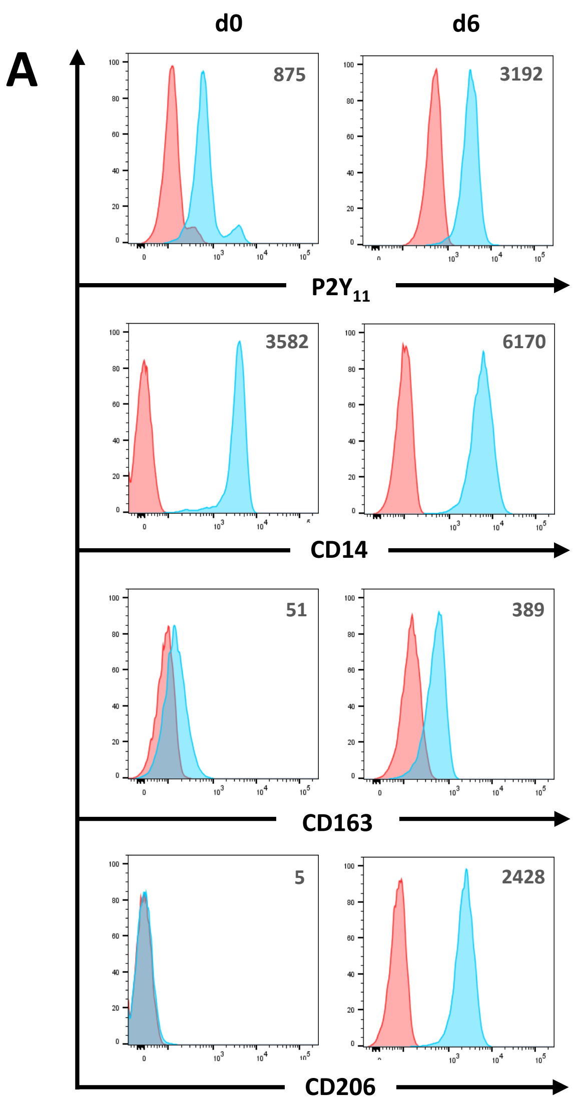

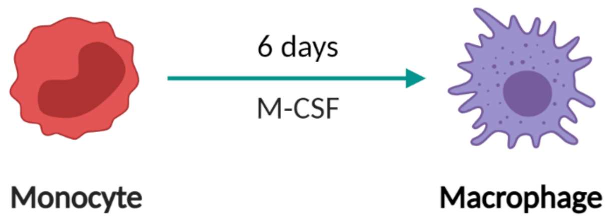

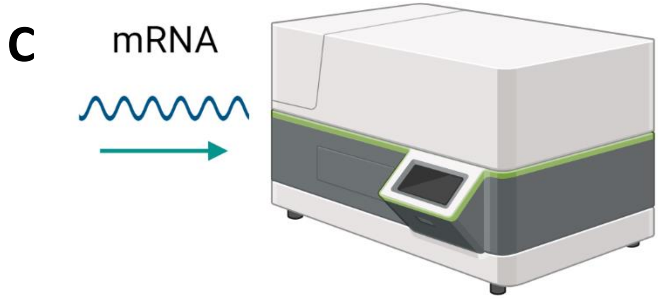

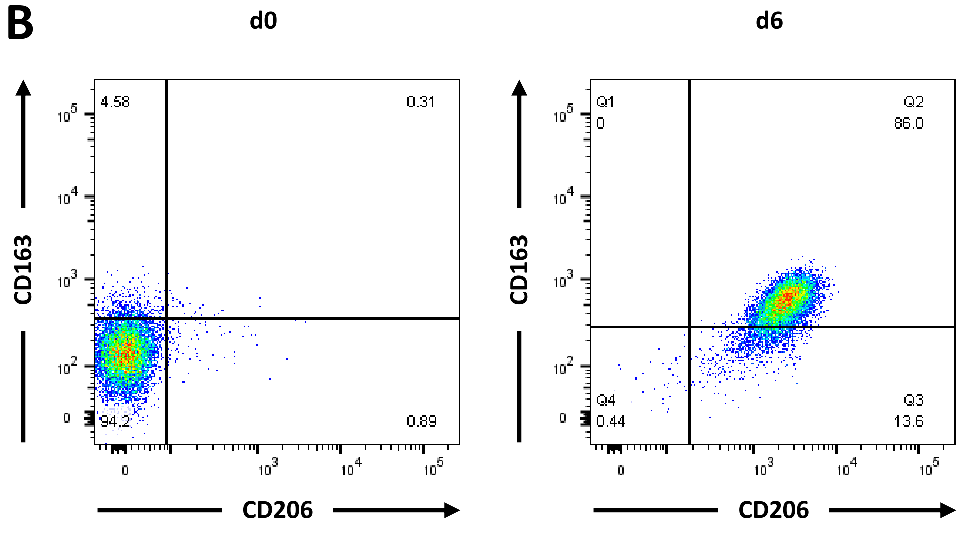

Fig. S1 Generation and characterization of M2-like macrophages derived from monocytes using M-CSF. **a** M2 macrophages were differentiated by culturing isolated monocytes in the presence of M-CSF (50 ng·ml^{-1}) for 6 days. The phenotype of M2 polarized macrophages was determined by flow cytometry. Numbers represent mean fluorescence intensities (MFIs) of the respective staining after subtraction of isotype control MFIs. **b** Co-expression of scavenger (CD163) and mannose (CD206) receptors was assessed to identify M2-like macrophages. **c** mRNA was isolated from macrophages and gene expression analysis was performed using NanoString technology.

Chemokine receptor	accession #	mRNA count	w/o Rolipram ATPγS / Ctrl	p value	ATPγS / ATPγS + NF340	p value	plus Rolipram ATPγS / Ctrl	p value	ATPγS / ATPγS + NF340	p value
**CCR1**	NM_001295.2	> 24.7	0,80	0,037	0,62	0,005	0,35	0,017	0,32	0,049
CCR2	NM_001123041.2	< 24.7	1,75	0,273	1,48	0,340	1,77	0,079	1,17	0,414
CCR3	NM_001837.2	< 24.7	1,10	0,819	1,06	0,886	1,09	0,405	1,41	0,477
CCR4	NM_005508.4	< 24.7	1,29	0,456	1,14	0,297	1,66	0,186	1,69	0,079
**CCR5**	NM_000579.3	> 24.7	0,74	0,023	0,55	0,034	0,42	0,001	0,41	0,076
CCR6	NM_031409.3	< 24.7	1,16	0,548	1,41	0,264	0,99	0,953	0,86	0,624
**CCR7**	NM_001838.3	> 24.7	1,82	0,094	1,13	0,505	2,23	0,040	1,41	0,184
CCR8	NM_005201.2	< 24.7	1,20	0,541	0,97	0,901	1,11	0,892	1,03	0,968
CCR9	NM_031200.1	< 24.7	0,89	0,807	0,95	0,899	1,14	0,726	1,59	0,291
CCR10	NM_016602.2	< 24.7	1,00	0,996	1,08	0,862	0,78	0,305	0,81	0,384
CXCR1	NM_000634.2	< 24.7	0,88	0,384	0,94	0,733	0,89	0,500	1,08	0,643
**CXCR2**	NM_001557.3	> 24.7	0,55	0,072	0,80	0,094	0,59	0,076	0,85	0,420
CXCR3	NM_001504.1	< 24.7	1,23	0,404	1,00	0,988	1,44	0,296	1,58	0,263
**CXCR4**	NM_001008540.1	> 24.7	**4,73**	**0,000**	**3,36**	**0,005**	**8,18**	**0,004**	**3,17**	**0,002**
CXCR5	NM_001716.4	< 24.7	1,55	0,316	1,53	0,276	1,92	0,188	1,43	0,177
**CXCR6**	NM_006564.1	> 24.7	1,12	0,702	1,31	0,427	1,37	0,292	1,04	0,785
ACKR2	NM_001296.5	< 24.7	1,46	0,324	1,42	0,227	1,34	0,586	0,92	0,828
**ACKR3**	NM_020311.1	> 24.7	**3,94**	**0,010**	**4,77**	**0,055**	**26,66**	**0,007**	**17,84**	**0,003**
**ACKR4**	NM_016557.2	> 24.7	0,40	0,068	0,52	0,123	0,38	0,009	0,72	0,172
**ACKR5**	NM_001130910.1	> 24.7	1,67	0,001	1,25	0,091	1,74	0,022	1,06	0,167

Table S1. Upregulation of CXCR4 and CXCR7 are hallmarks of a highly selective change in the chemokine receptor profile of macrophages in response to $P2Y_{11}$ activation. M2 macrophages were left untreated (Ctrl) or stimulated for 6 h with $P2Y_{11}$ receptor agonist ATPγS (20 µM) ± PDE4 inhibitor rolipram (10 µM). Copy numbers of CXCR7 mRNA were determined using NanoString technology. $P2Y_{11}$ receptor antagonist NF340 (20 µM) served to confirm that ATPγS-mediated changes were specific to $P2Y_{11}$ receptor activation. By using a set of negative control probes the general gene expression threshold was determined to be 24.7. **ATPγS/Ctrl**: fold increase induced by ATPγS over unstimulated cells (Ctrl); **ATPγS/ATPγS+NF340**: fold inhibition by $P2Y_{11}$ receptor antagonist NF340 relative to ATPγS-stimulated cells;

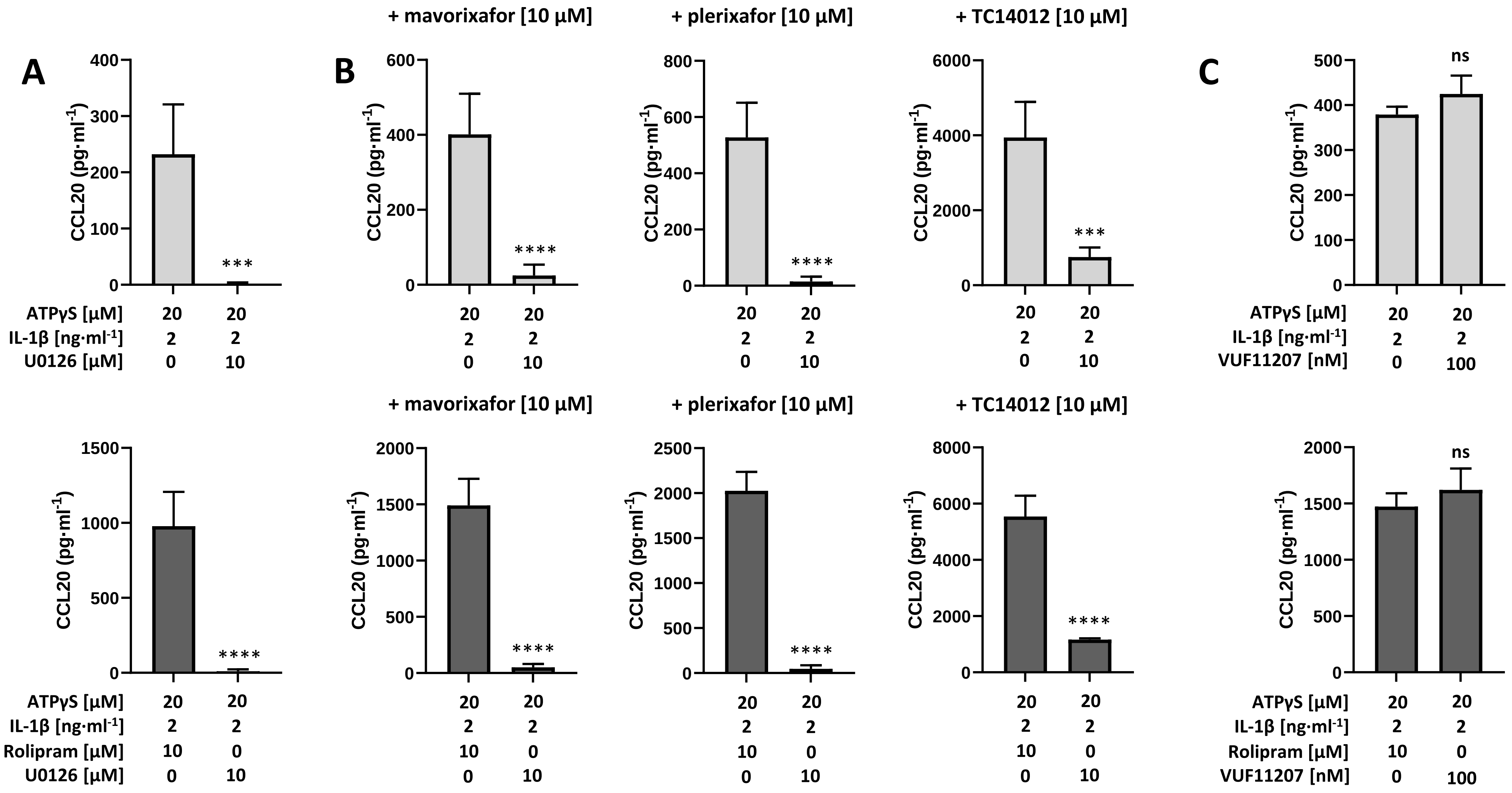

Fig. S2 CCL20 production induced by $P2Y_{11}$/IL-1R and enhanced by CXCR4 antagonists depends on MEK/ERK signaling in macrophages. **a** M2 macrophages were treated for 24 h with $P2Y_{11}$ receptor agonist ATPγS (20 µM) plus IL-1ß (2 ng/ml) in the absence (upper panel) or presence of PDE4 inhibitor rolipram (10 µM) (lower panel). U0126 was used to examine involvement of MEK/ERK signaling. **b** CXCR4 antagonists mavorixafor, plerixafor and TC14012 were used to modulate $P2Y_{11}$/IL-1R induced (upper panels) and rolipram-enhanced CCL20 production (lower panels). U0126 was used to examine involvement of MEK/ERK signaling (n = 3). **c** VUF11207, a CXCR7 agonist that is unable to activate ERK via ß-arrestin, was used as a control (n = 3 for all graphs). Mean values ± SD are shown. $***p < 0.001$, $****p < 0.0001$.

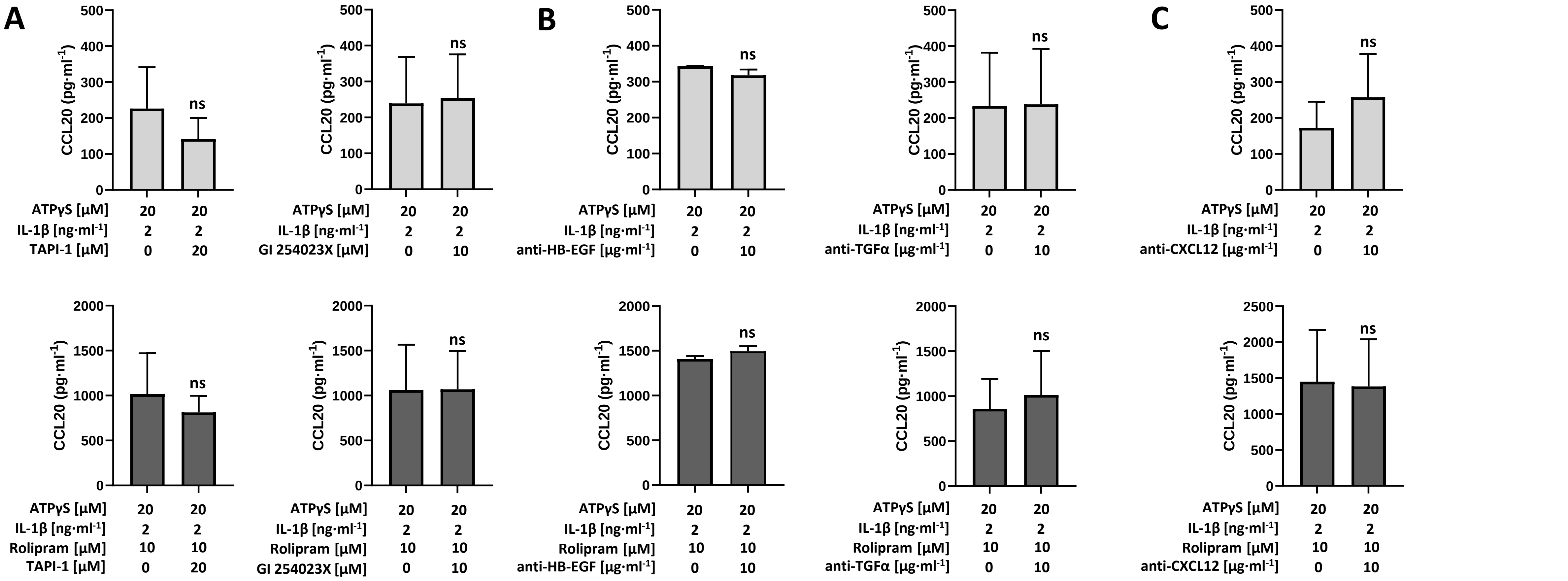

Fig. S3 P2Y$_{11}$/IL-1R induced and rolipram-enhanced CCL20 production appears to be independent of EGFR and CXCR7 ligands in macrophages. M2 macrophages were treated for 24 h with P2Y$_{11}$ receptor agonist ATPγS (20 µM) plus IL-1ß (2 ng/ml) in the absence (upper panel) or presence of PDE4 inhibitor rolipram (10 µM) (lower panel). **a** TAPI-1 and GI 254023X were used to examine a potential role of ADAM17 and ADAM10, respectively in the release (shedding) of EGFR ligands. **b** The effects of the neutralization of the EGFR ligands HB-EGF or TGF-α were studied. **c** The effects of the neutralization of the CXCR7 ligand CXCL12 were studied (n ≥ 3 for all graphs). Mean values ± SD are shown.

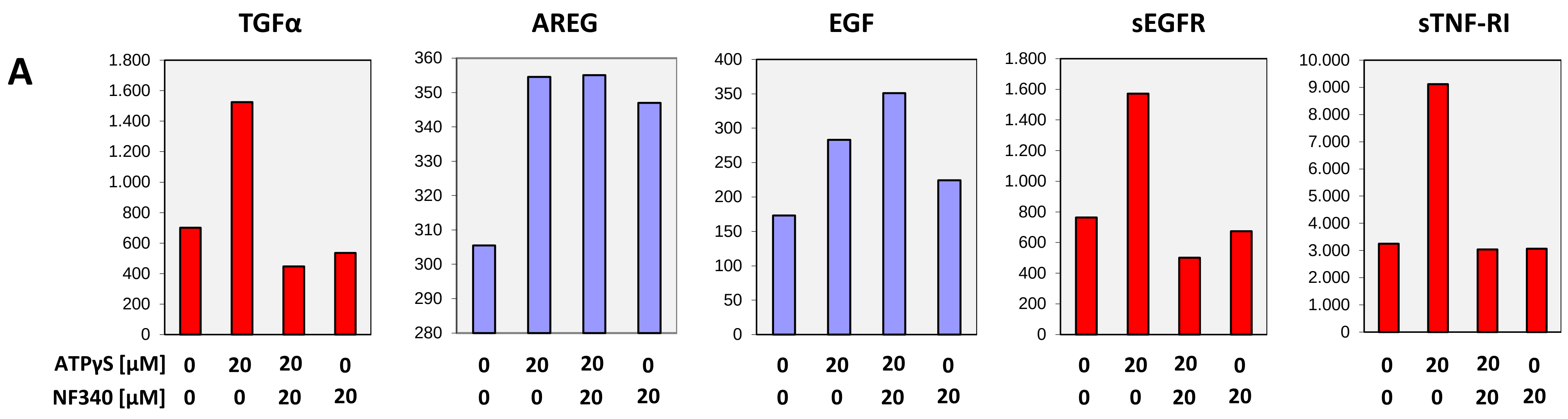

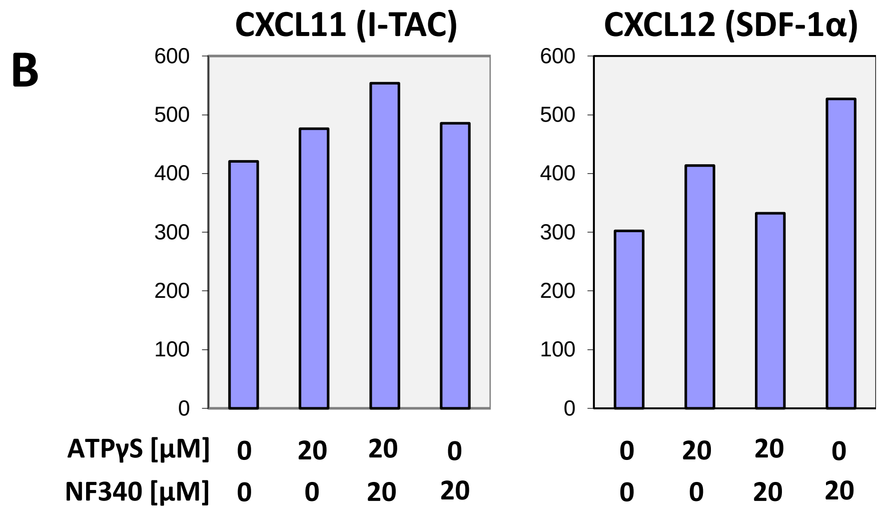

Fig. S4 Analysis of the astrocytoma secretome for the presence of EGFR and CXCR7 ligands. $P2Y_{11}$-recombinant astrocytoma cells were treated for 24 h with $P2Y_{11}$ receptor agonist ATPγS (20 µM). $P2Y_{11}$ receptor antagonist NF340 (20 µM) served to confirm that ATPγS-mediated changes were specific to $P2Y_{11}$ receptor activation. The Human Cytokine Antibody Array Kit G2000 was used to detect 174 cytokines. Y-axis: the G-Series arrays are semiquantitative featuring fluorescent signal detection (a.u., arbitrary units). **a** EGFR ligands: Tumor growth factor α (TGFα), amphiregulin (AREG), epidermal growth factor (EGF); ectodomain shedding of EGFR (sEGFR) indicating strong EGFR activation in malignant cells was also examined; ectodomain shedding of TNF-RI (sTNF-RI), which was previously shown to be a $P2Y_{11}$ target (PMID: 33463722) and occurs at much higher levels, is shown for control purposes. **b** CXCR7 ligands: CXCL11, also known as interferon-inducible T cell alpha chemoattractant (I-TAC); CXCL12, also known as stromal cell-derived factor 1α (SDF-1 α).

A

Receptor / Ligand	LOD pg/ml	MAX pg/ml	ATPγS + IL-1ß pg/ml	NF340 % inhibition
AR	35,1	10.000,0	31,1	0.6
EGF	0,1	37,0	0,0	0,0
HB-EGF	1,0	1.851,9	3,0	100,0
TGFα	1,5	1.851,9	63,3	100,0
EGFR	18,0	10.000,0	18,5	4,9
TNF-RII	142,2	40.000,0	14.343,8	83,4

B

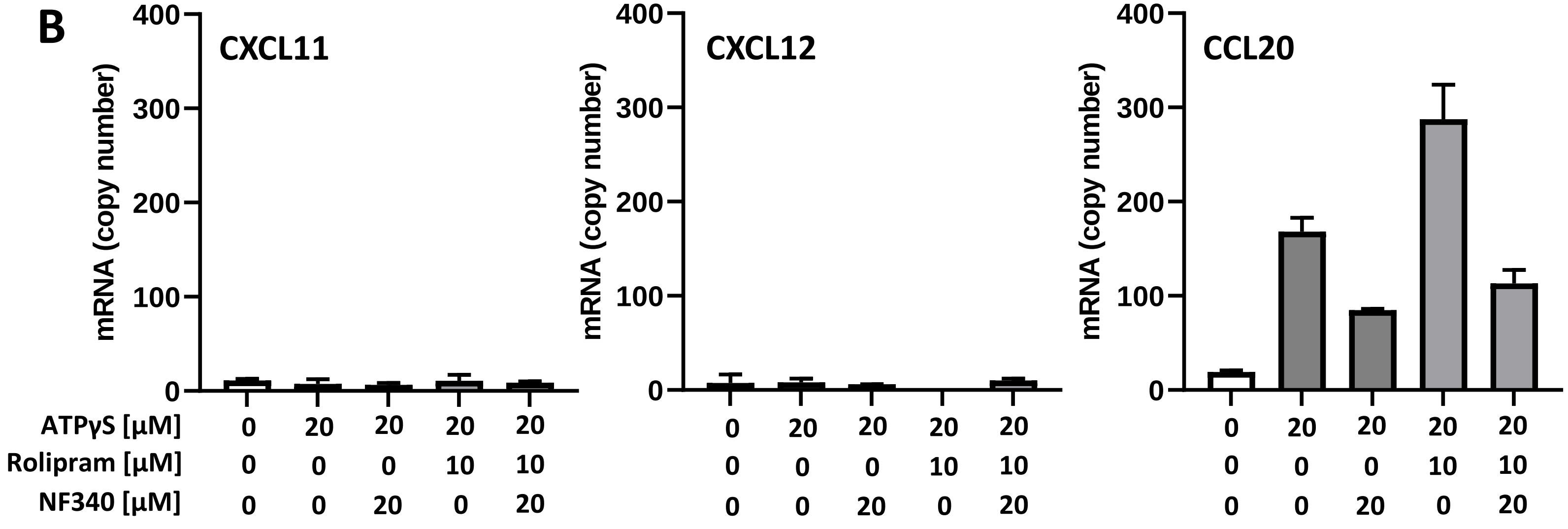

C

Chemokine	LOD pg/ml	MAX pg/ml	ATPγS + IL-1ß pg/ml	NF340 % inhibition
CXCL11	20,0	5.555,6	2,5	0,0
CXCL12	11,5	10.000,0	27,8	70,5
CCL20	0,3	246,9	59,7	83,2

Fig. S5 Analysis of the M2 macrophage secretome and transcriptome for the presence of EGFR and CXCR7 ligands. **a** EGFR ligands: M2 macrophages derived from three different donors were treated for 24 h with $P2Y_{11}$ receptor agonist ATPγS (20 µM) plus IL-1ß (2 ng/ml) in the presence or absence of either CXCR4 antagonist plerixafor (10 µM) or CXCR7 agonist TC14012 (10 µM). Pooled supernatants were analyzed for the presence of EGFR ligands using RayBio technology. Ectodomain shedding of TNF-RII (sTNF-RII), which was previously shown to be a $P2Y_{11}$ target (PMID: 33463722) and occurs at much higher levels, is shown for control purposes. **b** CXCR7 ligands (mRNA): M2 macrophages were treated for 6 h with $P2Y_{11}$ receptor agonist ATPγS (20 µM) ± PDE4 inhibitor rolipram (10 µM). Copy numbers of CXCR7 mRNA were determined using NanoString technology. $P2Y_{11}$ receptor antagonist NF340 (20 µM) served to confirm that ATPγS-mediated changes were specific to $P2Y_{11}$ receptor activation. CCL20, which was previously shown to be a $P2Y_{11}$ target (PMID: 36107259) and occurs at much higher levels, is shown for control purposes. Threshold: 24.7 **c** CXCR7 ligands (protein): pooled macrophage supernatants were analyzed for the presence of the CXCR7 ligands CXCL11 and CXCL12 using RayBio technology. CCL20, which was previously shown to be a $P2Y_{11}$ target, is shown for control purposes.

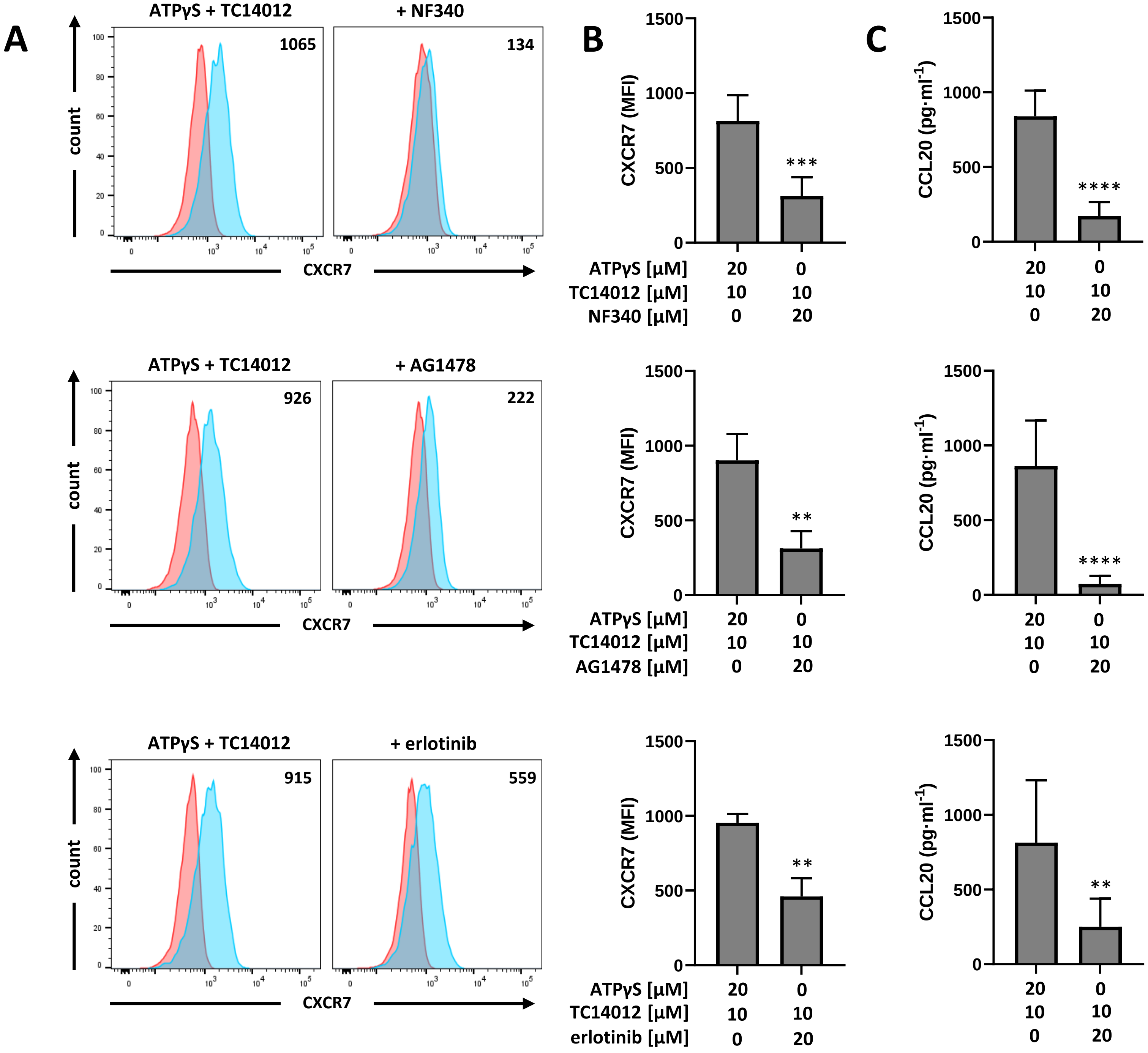

Fig. S6 Synergistic cooperation between $P2Y_{11}$ and CXCR7 in primary human macrophages depends on EGFR. **a** M2 macrophages were treated for 24 h with $P2Y_{11}$ receptor agonist ATPγS and CXCR7 agonist TC14012, alone or in combination. To examine EGFR involvement, EGFR-TKI AG-1478 (10 µM) and erlotinib (20 µM) were used to modulate CXCR7 expression and CCL20 production. For comparison, effects of $P2Y_{11}$ receptor antagonist NF340 are also shown. Representative FACS histograms of CXCR7 expression (light blue; isotype controls in red) are shown in the left panel. Numbers represent mean fluorescence intensities (MFIs) of the respective staining after subtraction of isotype control MFIs. **b,c** Quantification of CXCR7 expression and CCL20 production is shown in the right panel ($n \geq 3$ for all graphs). Data shown are mean values ± SD. $^{**}p < 0.01$, $^{***}p < 0.001$.

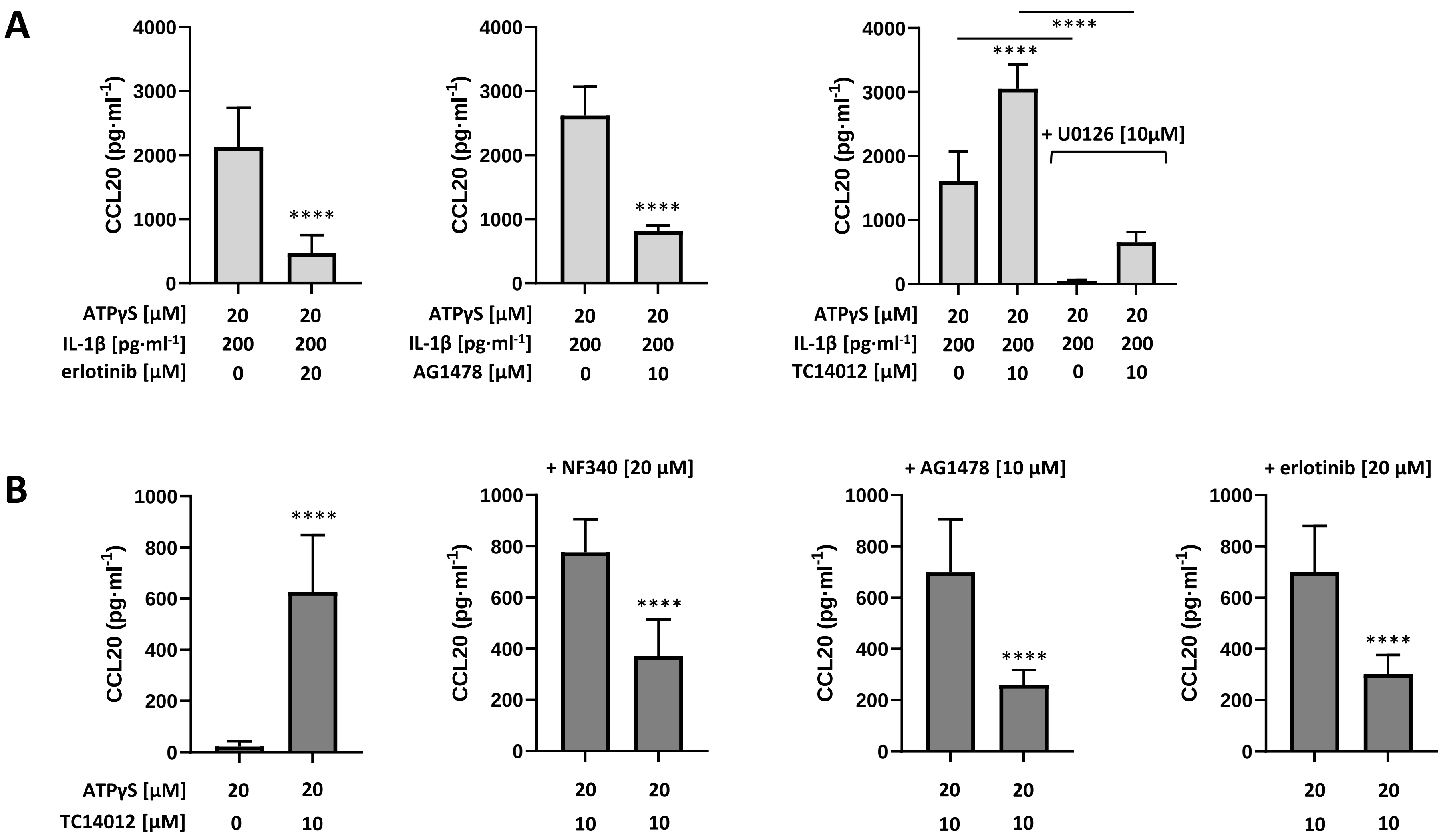

Fig. S7 P2Y$_{11}$/IL-1R- and P2Y$_{11}$/CXCR7-driven CCL20 production depends on EGFR in astrocytoma cells. **a** P2Y$_{11}$-recombinant astrocytoma cells were treated for 24 h with P2Y$_{11}$ receptor agonist ATPγS alone or in combination with IL-1ß. **b** M2 macrophages were treated for 24 h with P2Y$_{11}$ receptor agonist ATPγS alone or in combination with CXCR7 agonist TC14012. CCL20 was measured in culture supernatants. **a,b** To examine EGFR involvement, the EGFR-TKIs AG-1478 and erlotinib were used to modulate CCL20 production. **c** U0126 was used to examine involvement of MEK/ERK signaling (n ≥ 3 for all graphs). Data shown are mean values ± SD. ****$p < 0.0001$.

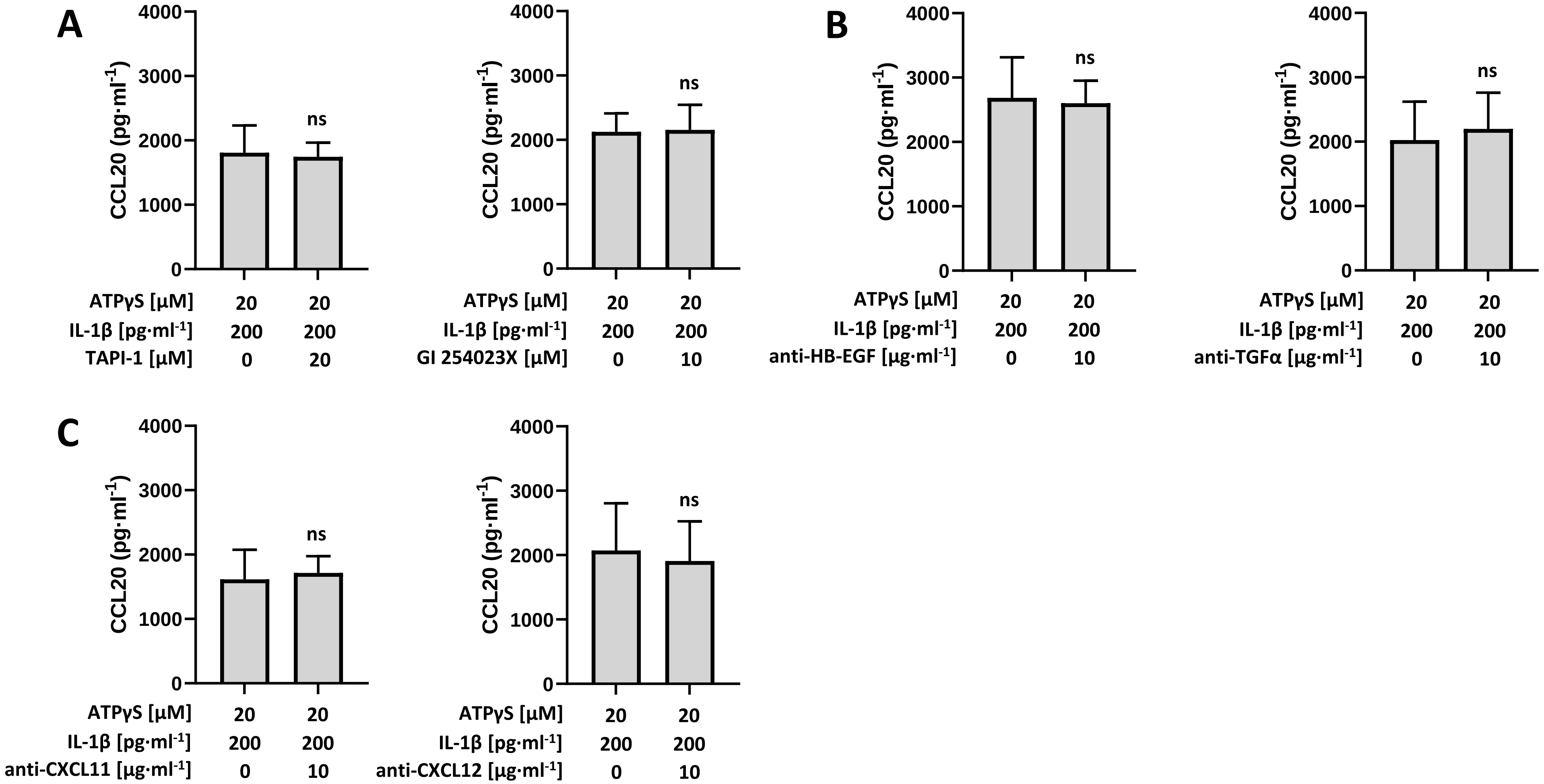

Fig. S8 $P2Y_{11}$/IL-1R induced and rolipram-enhanced CCL20 production appears to be independent of EGFR and CXCR7 ligands in astrocytoma cells. $P2Y_{11}$-recombinant astrocytoma cells were treated for 24 h with $P2Y_{11}$ receptor agonist ATPγS (20 µM) plus IL-1ß (2 ng/ml). **a** TAPI-1 and GI 254023X were used to examine a potential role of ADAM17 and ADAM10, respectively in the release (shedding) of EGFR ligands. **b** The effects of the neutralization of the EGFR ligands HB-EGF or TGF-α were studied. **c** The effects of the neutralization of the CXCR7 ligands CXCL11 and CXCL12 were studied (n = 3 for all graphs). Data shown are mean values ± SD.

Fig. S9 Analysis of CCL chemokines in the P2Y$_{11}$/IL-1R activated macrophage secretome: modulation by NF340, TC14012 and plerixafor.

a M2 macrophages derived from three different donors were treated for 24 h with P2Y$_{11}$ receptor agonist ATPγS plus IL-1ß in the presence or absence of either CXCR4 antagonist plerixafor or CXCR7 agonist TC14012. Pooled supernatants were analyzed for the presence of 20 CCL chemokines using RayBio technology. Mean values ± SEM calculated from the 3 donors are shown for comparison. $**p < 0.05$, $***p < 0.001$, $****p < 0.0001$ **b** CCL chemokines present in the macrophage secretome but resistant to inhibition with NF340. **c** CCL chemokines present in the macrophage secretome and sensitive to inhibition with NF340.

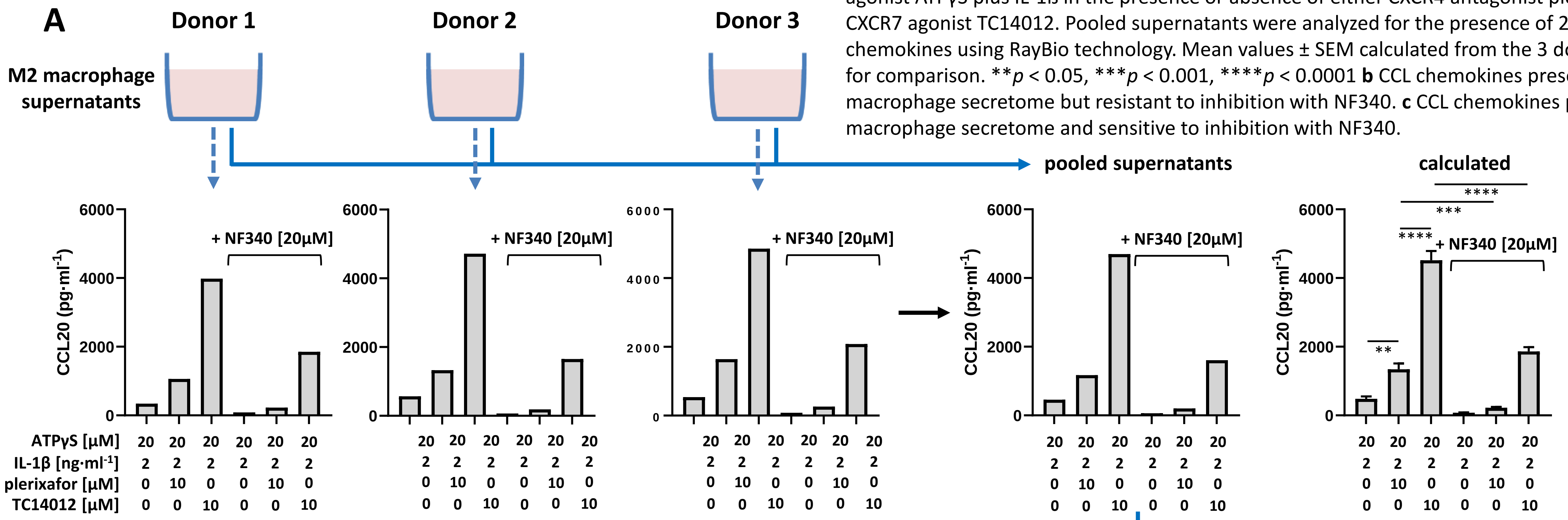

Undetectable: CCL11, CCL14, CCL15, CCL16, CCL17, CCL19, CCL27

B

Chemokine	Synonym	LOD pg/ml	MAX pg/ml	ATPγS + IL-1ß pg/ml	NF340 % inhibition
CCL1	I-309	8,0	4.000,0	240,8	0,0
CCL5	RANTES	59,0	20.000,0	264,5	0,0
CCL8	MCP-2	0,5	370,4	109,5	0,0
CCL13	MCP-4	4,9	5.555,6	195,8	0,0
CCL18	PARC	8,0	2.222,2	608,3	0,0
CCL23	MPIF-1	20,0	10.000,0	584,3	0,0
CCL24	Eotaxin-2	7,1	1.000,0	168,6	0,0

C

Chemokine	Synonym	LOD pg/ml	MAX pg/ml	ATPγS + IL-1ß pg/ml	NF340 % inhibition
CCL2	MCP-1	3,9	1.111,1	1.117,5	37,0
CCL3/L1/L3	MIP-1α	33,7	10.000,0	5.205,5	40,9
CCL4/L1/L2	MIP-1ß	3,7	1.000,0	1.196,3	**59,7**
CCL7	MCP-3	10,1	4.000,0	4.369,1	49,5
**CCL20**	MIP-3α	0,3	246,9	59,7	**83,2**
CCL21	6Ckine	80,0	40.000,0	2.011,9	**74,1**
CCL22	MDC	1,3	1.851,9	724,9	33,5
CCL25	TECK	84,5	18.518,5	317,3	29,5
CCL26	Eotaxin-3	40,0	20.000,0	107,7	72,2
CCL28	MEC	80,0	40.000,0	312,3	40,4
